# Cell signaling and transcriptional regulation of osteoclast lineage commitment, differentiation, bone resorption and diseases

**DOI:** 10.1038/s41421-025-00853-6

**Published:** 2026-01-20

**Authors:** Siyu Zhu, Ming-Qi Yan, Alasdair Masson, Wei Chen, Yi-Ping Li

**Affiliations:** Division in Cellular and Molecular Medicine, Department of Pathology and Laboratory Medicine, Tulane University School of Medicine, Tulane University, New Orleans, LA USA

**Keywords:** Cell signalling, Cell growth

## Abstract

Osteoclasts are bone-resorbing cells that play a central role in normal bone remodeling and contribute to bone loss associated with pathological conditions such as osteoporosis, osteoarthritis, rheumatoid arthritis, periodontal disease, and bone metastases of cancer. The commitment, differentiation, and function of osteoclasts depend on the establishment of specific gene expression patterns orchestrated through a network of transcription factors, which are sequentially activated by osteoclastogenic signals. This review provides an updated overview of the roles of key signaling pathways (e.g., RANKL signaling, NF-κB signaling and Gα_13_ signaling), transcription factors (e.g., PU.1, C/EBP-α, NFATc1 and IRF8), cytokines (e.g., TNF-α, IL-1β and IL-6), and epigenetic regulators (e.g., DNMT3a, EZH2 and ASXL1) in osteoclast lineage commitment, differentiation and bone resorption under both physiological and pathological inflammatory conditions, along with insights from corresponding mouse models. We described the mechanism by which osteoclast-mediated bone resorption occurs through extracellular acidification driven by osteoclast-specific proton pump subunits (e.g., ATP6i and ATP6v0d2), followed by matrix protein degradation mediated by cathepsin K and MMP-9. Additionally, this review examines the interplay among molecular mechanisms that regulate osteoclast differentiation and activation under pathological and inflammatory conditions, elucidates their roles in osteoclast hyperactivation-related human diseases, and provides a comprehensive framework for understanding these processes. Finally, it underscores potential novel therapeutic strategies for osteoclast-related skeletal lytic diseases and highlights perspectives for future investigations.

## Introduction

Bone is a mineralized tissue that serves critical mechanical and metabolic functions^1^. Bone structure undergoes continuous remodeling throughout life in response to physiological and environmental cues. The coordinated and balanced activities of bone-forming osteoblasts and bone-resorbing osteoclasts drive the continuous turnover of bone tissue, a process referred to as bone remodeling. Bone undergoes continuous remodeling throughout life, and disruption of this balance leads to skeletal disorders. Osteoclasts, derived from the monocyte/macrophage lineage, work in coordination with osteoblasts to maintain bone health, increasing their resorptive activity when triggered by environmental signals^2–4^.

Osteoporosis, the most prevalent bone disorder, affects ~28 million adults in the United States and arises from elevated osteoclast number and activity, resulting in reduced bone mass and heightened fracture risk from minimal trauma^5^. In contrast, impaired osteoclast differentiation or dysfunction causes osteopetrosis, a rare disease marked by excessive bone mass and obliteration of the marrow cavity. While the receptor activator of nuclear factor kappa B ligand (RANKL)-receptor activator of nuclear factor kappa B (RANK)-osteoprotegerin (OPG)^6^ signaling axis, pro-inflammatory cytokines (e.g., tumor necrosis factor-α (TNF-α), interleukin-1 (IL-1)^7^, interleukin-6 (IL-6)^8^) and multiple metabolic pathways have been comprehensively discussed in previous reviews, the present article emphasizes the transcriptional and epigenetic mechanisms governing osteoclast differentiation. In particular, we highlight how lineage-determining and pioneer transcription factors, such as PU.1, interferon regulatory factor 8 (IRF8), and nuclear factor of activated T-cells, cytoplasmic 1 (NFATc1)^9^, which coordinate chromatin remodeling and gene expression during the distinct stages of osteoclastogenesis^10,11^.

Recent advances in seq-based omics technologies like RNA-seq, ChIP-seq, ATAC-seq, scRNA-seq and transcriptomics have enabled researchers to uncover new regulatory layers that govern osteoclast lineage specification and function. By focusing on the transcriptional logic and chromatin landscape that define osteoclast identity, we aim to provide a deeper mechanistic understanding of how specific transcription factor networks orchestrate osteoclastogenesis. These insights may help identify novel therapeutic targets for modulating osteoclast activity in bone diseases.

Several excellent reviews have recently summarized broader aspects of osteoclast biology. For example, Takegahara et al.^12^ provided an updated overview of osteoclast differentiation and maturation with particular emphasis on metabolic adaptation and therapeutic implications; Sun et al.^13^ comprehensively discussed osteoclast origin, differentiation, apoptosis, and coupling with osteoblasts; Zheng et al.^14^ highlighted the role of NFATc1 in rheumatoid arthritis (RA)-related bone destruction; and Veis offered an authoritative perspective on osteoclast function as key sculptors of bone^15^. Building on these important contributions, the present review specifically focuses on transcription factors, signaling pathways, and epigenetic regulators that orchestrate osteoclast differentiation and function. By concentrating on lineage-determining transcription factors, chromatin remodeling factors, and their crosstalk with intracellular signaling, we aim to provide an update mechanistic framework that complements recent reviews and offers new insights into osteoclast biology under both physiological and pathological conditions.

## Osteoclast origin, lineage and longevity

### Osteoclast lineage

**Fig. 1:**
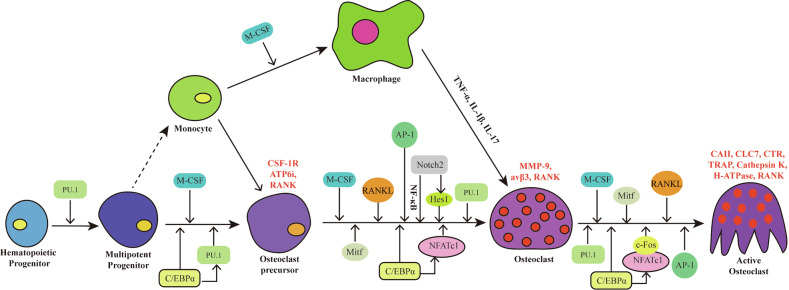
Different transcription factors regulate osteoclast differentiation fates. This figure illustrates the main differentiation stages of osteoclasts from the hematopoietic progenitors, highlighting the key signaling molecules, transcription factors, and cellular interactions involved in bone remodeling. The process begins with a hematopoietic progenitor cell, which, under the influence of PU.1, differentiates into a myeloid progenitor. The myeloid progenitor, stimulated by M-CSF, expresses C/EBPα and differentiates into a monocyte. Monocytes, upon receiving signals from M-CSF, can further differentiate into macrophages. Alternatively, in the presence of M-CSF and RANKL, and driven by factors like PU.1, C/EBPα, MITF, and AP-1, the monocyte can differentiate into OCP. The OCP, upon additional signaling from M-CSF and RANKL, differentiates into a multinucleated osteoclast with further involvement of NFATc1 and c-Fos. The mature osteoclast, which can be activated by signals like MMP-9 and p38, expresses enzymes such as carbonic anhydrase II (CAII), CTR, TRAP, CTSK, and H^+^-ATPase, leading to bone resorption.

Osteoclast lineage commitment, phenotype, and gene expression are fundamentally linked to their differentiation process. Transplantation studies have established that osteoclast precursors (OCPs) are mononuclear cells of hematopoietic origin^16^. Osteoclasts are specialized, well-defined cells of the bone marrow that originate from myeloid progenitors or bone marrow-derived macrophages (BMDMs) and are primarily responsible for bone resorption (Fig. 1). Circulating mononuclear precursors migrate to bone surfaces, where they fuse under specific stimuli to generate mature multinucleated osteoclasts. Within the marrow niche, progenitors can differentiate into either osteoclasts or bone-resident macrophages depending on external cues, and bone macrophages themselves retain the capacity to further differentiate into osteoclasts (Fig. 1). More broadly, macrophages are mononuclear immune cells distributed across most tissues, where they function in host defense, regulation of inflammation, and clearance of apoptotic cells and debris. In mice, bone-resident macrophages typically constitute ~15–20% of total bone marrow cellularity^17^.

OCPs are characterized by the presence of multiple Golgi apparatuses, abundant mitochondria^18^, and positive staining for non-specific esterase^19^, and in certain cases, type IV collagenase. Under appropriate microenvironmental conditions, these precursors further mature into pre-osteoclasts^20^. Pre-osteoclasts are tartrate-resistant acid phosphatase (TRAP)-positive cells that express mRNAs characteristic of osteoclast lineage and, upon further differentiation, fuse to form multinucleated mature osteoclasts (Fig. 1)^21,22^. MOESIN, a cytoskeletal linker protein of the Ezrin/Radixin/Moesin (ERM) family, plays a crucial role in this process, the inhibition of which enhances osteoclast fusion by reducing membrane-to-cortex attachment and promoting tunneling nanotube formation, while also increasing osteoclast activity and bone resorption by regulating the sealing zone formation via the β3-integrin/RhoA/SLK pathway^23^. Sorting nexin 10 (SNX10) is also essential for regulating the fusion process^24^. Osteoclasts deficient in SNX10 show the prolonged presence of DC-STAMP protein in the periphery, which may contribute to their uncontrolled fusion^24^. Uncontrolled fusion of osteoclasts results in the formation of giant inactive osteoclasts that are, on average, 2–6 times larger in size. NINEIN, a centrosomal protein, has also been implicated in osteoclast fusion by regulating centrosomal microtubule organization, promoting centrosome cohesion and clustering in multinucleated mature osteoclasts, the disruption of which leads to a premature ossification phenotype^25^.

Osteoclasts possess a specialized proton-pumping system that enables efficient dissolution of mineralized bone. In parallel, they secrete collagenases^26^, cathepsin K (CTSK)^27^, and various hydrolases, which together facilitate degradation of the organic components of the bone matrix. In vitro osteoclastogenesis can be replicated using several models, including organ cultures of murine embryonic metatarsals^28^, co-culture of hematopoietic progenitors from spleen or bone marrow with osteoblasts^29^, or immortalized precursor cell lines such as MOCP-5^30^. Furthermore, treatment with soluble RANKL and macrophage colony-stimulating factor (M-CSF) alone is sufficient to drive spleen- or marrow-derived precursors to differentiate into mature osteoclasts, even in the absence of osteoblastic support^31^.

It is well known that osteoclasts are differentiated from hematopoietic stem cells (HSCs) (Fig. 1). A recent study indicates that most osteoclasts in neonatal bone originate from yolk sac-derived macrophages of erythro-myeloid progenitor (EMP) lineage^32^. These macrophages can fuse with HSC-derived precursors during osteoclastogenesis^32^. Notably, Cx3cr1^+^ yolk sac-derived macrophages develop independently of the HSC lineage and generate long-lived osteoclasts, whereas Csf1r^+^ progenitors lack this capacity^32^. It has been proposed that a distinct Cx3cr1^+^ cell population present at embryonic day 9.5 contributes to the formation of long-lasting osteoclasts^32^. Functionally, EMP-derived osteoclasts are indispensable for bone cavity development and facilitate HSC colonization of the skeletal niche. Furthermore, another subset of EMP-derived macrophages persists beyond 0.5 months of age in mice, continuing to provide OCPs, thereby suggesting the existence of long-term osteoclast populations in adulthood^32^.

These findings elucidate the complex origins and developmental plasticity of osteoclasts, whose formation is influenced by both embryonic and hematopoietic sources. While lineage commitment and intracellular signaling are key to osteoclastogenesis, their differentiation is also critically regulated by the local bone microenvironment. Within this microenvironment, direct interactions with neighboring cell types, particularly osteoblasts and stromal cells, play a crucial role in fine-tuning osteoclast development, fusion, and resorptive activity.

### Cell–cell interaction effects on osteoclast differentiation

In addition to lineage-intrinsic mechanisms, osteoclast differentiation is also influenced by extrinsic regulatory factors within the bone microenvironment, particularly through direct interactions with neighboring cell types such as osteoblasts. Osteoclasts and osteoblasts are closely regulated through mutual, bidirectional signaling, with pathways such as Ephrin signaling playing a key role in their differentiation. Ephrins and their corresponding Eph receptors are expressed in bone cells as well as their progenitors, establishing a signaling axis that plays a crucial role in regulating the differentiation of both osteoclasts and osteoblasts. This pathway is regulated by transmembrane ligands such as Ephrin2 and receptor tyrosine kinases including EphB4 (Fig. 1). The osteoblasts inhibit osteoclast formation through EphB4 signaling, while simultaneously promoting osteoblast differentiation via Ephrin-B2^33^. EphB4-mediated “forward” signaling from osteoclasts to osteoblasts enhances osteoblast differentiation and bone formation, whereas “reverse” signaling through Ephrin-B2 from osteoblasts to osteoclasts inhibits osteoclast activity, thus reducing bone resorption^34^. In addition, it was suggested that EphrinB2 signaling inhibited osteoclast formation by blocking the induction of Fos induced by RANKL and its transcriptional target NFATc1. Additionally, EphB4 signaling is known to induce the expression of important regulators of osteoblast differentiation, such as Dlx5, Osx, and Runx2^35^. A recent study reported that when gingival fibroblasts undergoing osteogenic differentiation were co-cultured with OCPs, the formation of osteoclasts was reduced, which was associated with a decrease in TNF-α secretion^36^.

Eosinophils, which are located near bone-resorbing osteoclasts in bone marrow, are involved in tissue homeostasis. Loss of eosinophils in ΔdblGATA mice leads to decreased bone mass at baseline and exacerbated bone loss following sex hormone deprivation or inflammatory arthritis^37^. In interleukin-5 (IL-5) transgenic mice, elevated eosinophil levels promote bone mass under homeostatic conditions and confer protection against hormone- or inflammation-induced bone loss. Eosinophils potently suppress osteoclast differentiation and resorptive activity, while markedly altering osteoclast transcriptional programs^37^. The osteoclast-inhibitory effect of eosinophils is based on the release of eosinophil peroxidase, leading to damage by reactive oxygen species (ROS) and induction of mitogen-activated protein kinases (MAPKs) in OCPs^37^.

The crosstalk between osteoclasts and M2 macrophages is important in restoring the balance between osteoclasts and M2 macrophages^38^. M2 macrophage-derived extracellular vesicles, containing the molecular metabolite glutamate, can downregulate osteoclast-specific gene expression and convert OCPs to M2 macrophage-like lineage cells^38^. Upon glutamate uptake, glutamine metabolism in OCPs is significantly upregulated, leading to increased production of α-ketoglutarate^38^. This metabolite facilitates Jumongi-containing 3 domain (JMJD3)-dependent epigenetic reprogramming, driving M2-like macrophage differentiation^38^.

### Osteoclast recycling and longevity

Beyond differentiation and inhibition, recent evidence suggests that the fate of osteoclasts is far more dynamic than previously believed. Rather than undergoing terminal apoptosis after resorption, osteoclasts may persist through mechanisms of cellular recycling and functional reprogramming. Among these emerging concepts, the discovery of osteoclast recycling has introduced a fundamentally new perspective on osteoclast longevity and function. The concept of osteoclast recycling marks a fundamental shift in our understanding of osteoclast biology, overturning the traditional view that osteoclasts invariably undergo apoptosis after completing bone resorption. Recent studies have shown that osteoclasts can undergo fission into smaller, functional daughter cells known as osteomorphs, which are transcriptionally distinct from mature osteoclasts. These osteomorphs circulate in the blood and bone marrow and can later re-fuse to form active resorbing osteoclasts when stimulated by RANKL^39^. This recycling mechanism significantly extends the lifespan of osteoclasts, enabling them to participate in multiple cycles of bone resorption. Furthermore, osteoclast recycling is tightly regulated by RANKL signaling, with the inhibition of RANKL leading to the accumulation of osteomorphs and their precursors. Upon cessation of RANKL inhibition, these primed cells can rapidly reform active osteoclasts, contributing to the rebound phenomenon observed in bone loss following treatments like denosumab. This novel understanding of osteoclast recycling provides insights into bone resorption dynamics and highlights potential therapeutic avenues for regulating osteoclast activity in skeletal diseases.

Recent advancements in osteoclast biology have revealed a paradigm shift in understanding their lifecycle. Previously considered terminal cells undergoing apoptosis after a single resorption cycle, osteoclasts are now known to exhibit recycling capabilities through a process of fission into smaller cells termed osteomorphs. These osteomorphs can circulate, re-fuse, and form active osteoclasts upon stimulation, significantly extending their lifespan^32,40–43^. This process has critical implications for understanding the rebound effect observed in bone loss following anti-resorptive therapy discontinuation, such as with denosumab, where accumulated osteomorphs rapidly reassemble into resorbing osteoclasts^40,44–46^.

## Signaling pathways in osteoclast differentiation and function

**Fig. 2:**
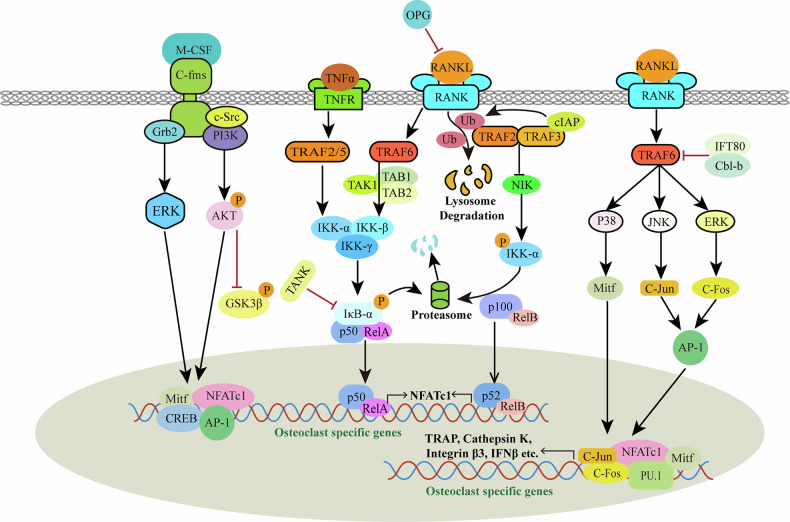
Canonical signaling pathways in osteoclast differentiation. This figure depicts the intricate signaling networks that govern osteoclast differentiation and activation, focusing on pathways initiated by M-CSF, RANKL, and TNF-α. The figure highlights the downstream signaling cascades, transcription factors, and target genes involved in osteoclastogenesis and function. The binding of M-CSF to its receptor c-Fms activates downstream signaling through c-Src and PI3K. This leads to the activation of AKT, which inhibits GSK3β, thereby stabilizing β-catenin and promoting osteoclast survival. Additionally, M-CSF triggers the ERK pathway via Grb2 and ERK, leading to the activation of AP-1 transcription factors, such as MITF and CREB, which upregulate osteoclast-specific genes. RANKL binding to RANK activates several downstream pathways, including the TRAF6-mediated NF-κB Pathway. TRAF6 interacts with TAB1/TAB2 to activate the TAK1 complex, which subsequently activates the IKK complex (IKKα, IKKβ, IKKγ), leading to the phosphorylation and degradation of IκBα. This allows the translocation of p50/RelA or p52/RelB complexes to the nucleus, driving the expression of osteoclast-specific genes, such as those encoding TRAP, CTSK, and integrin β3. TRAF6 also leads to the activation of NFATc1 through pathways involving p38, MITF, c-Jun, c-Fos, and AP-1, which synergistically enhance the transcription of osteoclast-specific genes. OPG acts as a decoy receptor for RANKL, preventing RANK activation and downstream signaling, thereby inhibiting osteoclast differentiation. TNF-α binds to its receptor TNFR, activating TRAF2/5 and TRAF6. These pathways converge on TAK1, which activates both IKK and JNK pathways, promoting the nuclear translocation of NF-κB components (p50/RelA) and the activation of AP-1 transcription factors, respectively. The NIK pathway is activated through TRAF3 degradation, which contributes to the processing of p100 to p52, enhancing NF-κB signaling. RANKL and M-CSF signaling pathways converge to regulate the expression and activity of transcription factors like NFATc1, c-Fos, and AP-1, which are crucial for osteoclast differentiation and function. The figure also illustrates the role of cIAP in ubiquitination and lysosome degradation processes, which are involved in regulating these signaling pathways.

Osteoblasts, chondrocytes and their mineralized matrix, stromal cells, and endothelial cells collectively create the niche that supports OCP recruitment. Among them, osteoblasts secrete key cytokines such as M-CSF and RANKL, which drive and regulate the differentiation of these precursors into mature osteoclasts (Fig. 2). M-CSF interacts with its receptor C-FMS on OCPs, promoting their survival and proliferation, while the RANK-RANKL and M-CSF signaling axes serve as the principal regulators of osteoclastogenesis. Downstream, activation of the nuclear factor kappa B (NF-κB) ligand pathway, a central mediator of RANK-RANKL signaling, plays a pivotal role in orchestrating osteoclast differentiation^47^. These pathways not only control osteoclast differentiation but also ensure their functionality in resorbing bone, highlighting the complexity of the signaling network involved in osteoclastogenesis (Fig. 2).

Negative regulatory mechanisms play an essential role in preventing excessive osteoclast activity, which can lead to pathological bone resorption and diseases like osteoporosis. Intrinsic cellular pathways and external factors like hormones and cytokines are critical in balancing osteoclast differentiation and activation. The interplay between these positive and negative signals forms a tightly regulated system that determines the lifespan, resorptive activity, and overall contribution of osteoclasts to bone remodeling. Dysregulation of these pathways can disrupt this balance, resulting in conditions such as osteoporosis or osteopetrosis, depending on whether there is excessive or insufficient osteoclast activity.

Understanding the signaling pathways that regulate osteoclastogenesis is crucial for developing therapeutic strategies. Key signaling cascades, including RANK-RANKL-NF-κB, bone morphogenetic protein (BMP), Notch, and Hippo pathways, drive osteoclast differentiation and activation, while inhibitory mechanisms, such as estrogen signaling and Gα_13_ pathways, suppress excessive bone resorption (Figs. 2 and 5). Many therapies for bone-related diseases revolve around inhibiting the NF-κB signaling pathway, including denosumab, a monoclonal antibody that inhibits RANKL; IKKβ inhibitors, which block the IκB kinase complex to reduce inflammation-induced bone loss; saracatinib, a Src kinase inhibitor targeting osteoclast activity; and bortezomib, a proteasome inhibitor used in multiple myeloma to slow bone resorption^48,49^. Exploring these pathways further offers valuable insights into the molecular framework governing osteoclastogenesis and its implications in bone diseases.

### Positive regulation of osteoclastogenesis

#### RANK signaling and TRAF adaptor proteins

Engagement of RANK by RANKL initiates intracellular signaling through the recruitment of tumor necrosis factor receptor-associated factors (TRAFs), which subsequently activate multiple downstream pathways, including MAPKs, NF-κB, Src, and Akt (Fig. 2)^50^. Among the TRAF family members associated with RANK signaling (TRAF1, -2, -5, and -6), TRAF6 functions as the pivotal adaptor essential for osteoclastogenesis^51,52^. RANK-mediated activation of NF-κB is indispensable for osteoclast formation in vitro, and disruption of TRAF6, an essential adaptor in this pathway, leads to severe osteopetrosis (Table 1)^51–57^. In contrast, TRAF2 and TRAF5 contribute only marginally to osteoclast differentiation^51,52^. Mechanistically, RANKL–RANK engagement recruits TRAF6, activating NF-κB signaling, inducing its nuclear translocation, and initiating transcription of osteoclast-specific genes^58,59^. Intraflagellar transport protein 80 (IFT80) regulates osteoclast differentiation by stabilizing Casitas B-lineage lymphoma proto-oncogene b (Cbl-b), a ubiquitin ligase that mediates TRAF6 degradation^60^. Silencing of IFT80 enhances the ubiquitination of Cbl-b and elevates TRAF6 expression, thereby amplifying RANKL-mediated activation of the NF-κB signaling axis and promoting excessive osteoclast formation^60^. Overexpression of IFT80 has been previously shown to reduce TRAF6 protein levels^60^. The Cbl family of adaptor proteins — including c-Cbl, Cbl-b, and Cbl-c — plays a vital role in osteoclast regulation. Specifically, c-Cbl primarily modulates osteoclast cytoskeletal organization and motility, whereas Cbl-b is responsible for controlling osteoclast population size^61^. The role of Cbl-c in osteoclasts remain largely unexplored. IFT80 could form a crucial bridge between TRAF6 and Cbl-b, ensuring proper degradation of TRAF6^60,62,63^. Loss of IFT80 disrupts this interaction, resulting in unregulated TRAF6 signaling and hyperactivated osteoclasts^60,62,63^. Besides, the functions of c-Cbl and Cbl-b are redundant in the regulation of osteoclast movement and survival^64^. These findings highlight the TRAF6/IFT80/Cbl-b axis as a key regulator of osteoclast differentiation and bone homeostasis.

**Table 1:** Established mouse models for osteoclast function: key genes involved in osteoclast differentiation and function.

Gene	Classification	Loss or gain of function/method/cre line	Phenotype	References
ATP6i (Atp6v0a3)	Proton pump subunit	Loss of function/KO/germline	Severe osteopetrosis due to the impaired extracellular acidification function	^276,355^
CTSK	Cysteine protease	Loss of function/KO/germline	Impaired degradation of bone matrix proteins; osteopetrosis	^273,356–359^
C/EBPα	Transcription factor	Loss of function/CKO/*CTSK-Cre*	Impaired osteoclast terminal differentiation, activation, and function; osteopetrosis	^183,185,360–362^
c-Fos	Transcription factor	Loss of function/KO/germline	A lineage shift from osteoclasts to macrophages; osteopetrosis.	^363–365^
c-Jun	Transcription factor	Loss of function/transgenic/germline	Impaired osteoclastogenesis due to the inhibition of RANKL-induced osteoclast differentiation; osteopetrosis.	^3,366–368^
		Loss of function/CKO/*Mx-Cre*		^90^
FHL2	Transcription corepressor	Loss of function/KO/germline	Enhanced osteoclast resorptive activity and RANKL-stimulated bone loss	^369^
Gna13	G protein subunit	Loss of function/CKO/*CTSK-Cre*	A drastic increase in both osteoclast number and activity (hyper-activation); osteoporosis.	^133,246^
		Loss of function/CKO/*LysM-Cre*		
		Gain of function/COE/*LysM-Cre*	Inhibit osteoclastogenesis; protect against bone loss	^246^
IKKβ	Serine/threonine kinase	Loss of function/KO/germline	Impaired RANKL-mediated NF-κB activation in osteoclast progenitors; prevention from inflammation-induced bone loss	^71^
IL-1	Cytokine	Loss of function/KO/germline	Inhibition of the activation of RANK signaling during osteoclastogenesis; increased femur mineral density, trabecular bone mass and cortical thickness	^267,370^
IRF8	Transcription factor	Loss of function/KO/germline	Severe osteoporosis due to increased numbers of osteoclasts, and enhanced bone destruction following LPS administration	^138,371^
M-CSF	Cytokine	Loss of function/KO/germline	Decrease in osteoclast number and impairment of bone resorption	^372–377^
MITF	Transcription factor	Loss of function/KO/germline	Osteopetrosis due to the lack of mature multinucleate osteoclasts	^378–380^
NFATc1	Transcription factor	Loss of function/CKO/*Mx-Cre*	Impaired differentiation and activation of osteoclasts; osteopetrosis	^381–383^
		Loss of function/CKO/*LysM-Cre*		
NF-κB1/2	Transcription factor	Loss of function/KO/germline	Osteopetrosis due to the lack of mature osteoclasts	^384–387^
PU.1	Transcription factor	Loss of function/KO/germline	Osteopetrosis due to the lack of osteoclasts	^155,388–391^
RANK	Receptor for RANKL	Loss of function/KO/germline	Profound osteopetrosis resulting from an apparent block in osteoclast differentiation	^231,233,392–395^
RGS10	Regulator of G protein signaling	Loss of function/KO/germline	Osteopetrosis and impaired osteoclast differentiation via disruption of RANKL-evoked RGS10/calmodulin-[Ca^2+^]_i_ oscillation-calcineurin-NFATc1 signaling pathway	^117,396–399^
RGS12	Regulator of G protein signaling	Loss of function/CKO/*Mx-Cre*	Impaired osteoclast differentiation and function with impaired Ca^2+^ oscillations and reduced NFAT2 expression; inhibition of pathological osteoclastogenesis and bone destruction	^354^
		Loss of function/CKO/*CD11b-Cre*		
TRAF6	Adaptor protein	Loss of function/KO/germline	Osteopetrosis with defects in bone remodeling and tooth eruption due to impaired osteoclast function; involved in IL-1, CD40 and LPS signaling	^53–57^

The NF-κB pathway operates through two distinct mechanisms: the canonical (classical) and noncanonical (alternative) signaling routes. In the canonical pathway, external stimuli trigger the IKK complex to phosphorylate and degrade IκB, thereby releasing and activating the p50/RelA heterodimer (Fig. 2)^65–67^. The IKK complex is composed of the catalytic subunits IKKα and IKKβ, together with the regulatory subunit IKKγ (NEMO)^68^. Both IKKα and IKKβ are essential for RANKL-induced osteoclastogenesis in vitro, whereas IKKi, though dispensable under normal physiological conditions, has been shown to be important for efficient osteoclast formation, its targeted deletion disrupts osteoclast development both in vitro and in vivo (Table 1)^69–71^. Although the specific function of IKKγ in osteoclasts has not been fully elucidated, mutations in the IKKγ gene have been reported in patients with X-linked recessive ectodermal dysplasia with immunodeficiency (EDA-ID) as well as in those with osteosclerosis accompanied by lymphedema (OL-EDA-ID)^72^. TRAF-associated NF-κB activator (TANK) acts as a negative regulator of osteoclastogenesis by suppressing NF-κB activation and preventing IκB phosphorylation, thereby restraining osteoclast differentiation^58^. It has been reported that Tank competes with TRAF2 as a negative regulator for receptor interactions^73^. TANK was also reported to inhibit RANK ubiquitination, which is essential for signal transduction on the RANK-TRAF6 axis^58^. Both may act as underlying TANK mechanisms that negatively affect osteoclast survival and function^58^. Monocyte chemoattractant protein-1 (MCP-5) inhibits osteoclast differentiation by upregulating Ccr5 expression and preventing RANKL-induced IκB degradation, thereby suppressing NF-κB activation^74^. Another study highlighted the efficacy of sulforaphane (SFX-01) in blocking the early stages of osteoclast differentiation via NF-κB signaling. SFX-01 downregulates DC-STAMP and modifies cytoskeletal architecture, exerting its effects through the NRF2 and NF-κB pathways^75^. Eltanexor is a selective inhibitor^76^ of nuclear export that covalently binds to and blocks the activity of exportin-1 (XPO1), the key protein responsible for mediating nuclear-to-cytoplasmic transport of critical regulatory factors. By inhibiting XPO1, Eltanexor prevents the export of tumor suppressors and growth-regulatory proteins such as p53, IκBα, and FOXO1, thereby restoring their nuclear function and contributing to growth suppression (Fig. 2)^77^. Selinexor (Sel) is also an inhibitor of XPO1, which specifically inhibits XPO1-mediated nuclear output and leads to nuclear accumulation of IκBα to inhibit NF-κB signaling^78^. Elt inhibited RANKL-induced osteoclast growth and activity in vitro in a dose-dependent manner^78^. Upon activation of the NF-κB signaling pathway, NF-κB dissociates from IκBα and translocates into the nucleus to regulate gene expression. However, continuous NF-κB activation can be suppressed by inhibiting its nuclear export through the use of XPO1 inhibitors, which block NF-κB shuttling between the nucleus and cytoplasm, leading to the nuclear accumulation of inactive NF-κB^78^. A recent study reported that ubiquitin-specific protease 18 (USP18), a deubiquitinating enzyme (DUB), suppresses the NF-κB signaling pathway to inhibit osteoclastogenesis, potentially targeting transforming growth factor beta (TGF-β)-activated kinase 1 (TAK1) or its upstream molecules^79^. This regulation may underlie the mechanism of hypoxia-induced osteoclast differentiation and the subsequent development of osteoporosis.

D-Limonene is a monoterpene found in citrus fruits, which can inhibit NF-κB activity and modulate bone cell differentiation^80^. In vitro studies showed that D-Limonene suppresses RANKL-induced osteoclast differentiation, and reduces osteoclastogenesis and bone resorption, while simultaneously promoting osteoblast differentiation and bone nodule formation^80^. Moreover, it significantly downregulates pro-osteoclastogenic cytokines, including IL-1β, TNF-α, and PTHrP, thereby decreasing osteoclast-promoting signals from osteoblasts^80^. These findings suggest that D-limonene may represent a natural compound capable of preserving or enhancing bone mass, with potential as a complementary therapeutic approach for osteoporosis^80^. Oleandrin, derived from the leaves of Nerium oleander, exhibits anti-inflammatory activity and influences osteoclast differentiation^81^. In vitro studies demonstrated that Oleandrin inhibits RANKL-induced osteoclast differentiation in a concentration-dependent manner, while in vivo experiment showed that it reduces bone loss in ovariectomized (OVX) mice^81^. The mechanism of action involves suppression of the MAPK and NF-κB signaling pathways, wherein Oleandrin interacts with the low-density lipoprotein receptor-related protein 4 (LRP4) on osteoclasts, disrupting downstream pathway activation and consequently attenuating osteoclast formation. Evidence to date suggests that Oleandrin may hold therapeutic potential for osteoporosis, emphasizing the role of inflammatory signaling modulation in strategies for bone disease treatment^81^.

Noncanonical NF-κB activation involves NF-κB-inducing kinase (NIK)-mediated processing of the p100 precursor into its active form, p52. NF-κB2, in turn, modulates TNF-α-induced osteoclastogenesis (Fig. 2)^82^. Overexpression of p100, but not p52, inhibits osteoclast differentiation by preventing p100 conversion^82^. TRAF3 exerts a negative regulatory role in osteoclastogenesis by promoting NIK degradation via ubiquitination, thereby suppressing noncanonical NF-κB signaling^83–86^. During osteoclast differentiation, TRAF3 protein levels exhibit dynamic changes that parallel p100 expression: TNF-α upregulates TRAF3, whereas RANKL induces its degradation^87^. Silencing TRAF3 in OCPs prevents TNF-α-mediated p100 accumulation and enhances osteoclast formation, while TRAF3 overexpression directly suppresses RANKL-induced osteoclastogenesis^87^. These results indicate that TRAF3 restrains osteoclast differentiation by limiting p100 processing^82^. In vivo, conditional deletion of TRAF3 in bone marrow progenitors leads to elevated osteoclast formation and mild osteoporosis, further supporting its essential role in maintaining bone homeostasis through negative regulation of osteoclast development^88^.

Downstream of RANK, activation of p38 MAPKs directly phosphorylates signal transducer and activator of transcription 1 (STAT1), thereby regulating the transcription of multiple target genes^89^. In vitro studies further demonstrate that c-Jun N-terminal kinases (JNKs), together with their upstream kinase MKK7, are required for osteoclastogenesis^90,91^. JNK phosphorylates c-Jun to enhance its transcriptional activity, while RANK-induced extracellular regulatory kinase (ERK) signaling phosphorylates c-Fos, together driving activator protein-1 (AP-1) activation (Fig. 2).

GPR125 functions as a positive regulator of osteoclastogenesis by promoting key signaling cascades that are critical for osteoclast differentiation and activity. GPR125 appears to promote osteoclastogenesis by activating the AKT-NF-κB pathway, which supports OCP survival and function, and the MAPK pathway, which drives their proliferation and differentiation (Figs. 2 and 6)^92^. These pathways converge to facilitate the formation of osteoclasts, contributing to bone resorption processes in both normal bone remodeling and pathological conditions such as osteoporosis and RA. Moreover, GPR125 may modulate the expression of downstream transcription factors, including NFATc1, thereby promoting the maturation and functional activation of osteoclasts^93^.

Leucine-rich repeat-containing G protein-coupled receptor 4 (LGR4) negatively regulates RANK signaling by modulating membrane-to-endosome trafficking during the process of osteoclast differentiation^94^. Upon binding to RANKL, LGR4 undergoes endocytosis and localizes in RAB5-positive early endosomes, where it continues to regulate osteoclastogenesis^94^. LGR4 deficiency leads to enhanced early endosomal signaling, reduced inhibitory phosphorylation of GSK-3β, and increased nuclear translocation of NFATc1, ultimately promoting osteoclast differentiation^94^. These findings suggest that LGR4-mediated RANKL signaling is regulated by endosomal trafficking, providing insights into the intracellular mechanisms controlling osteoclastogenesis and bone resorption^94^.

Recent research explored the role of miR-19a in promoting osteoclast differentiation by inhibiting cylindromatosis (CYLD) expression. This suppression enhances the K63 ubiquitination of TRAF6, activating downstream NF-κB and MAPK signaling pathways critical for osteoclastogenesis (Fig. 2). The study establishes miR-19a as a key factor in pathological bone resorption, particularly in the context of pituitary adenoma-related bone invasion^95^. This study underscores the therapeutic potential of antioxidants in controlling excessive osteoclast activity^75^. Proteomic analysis has also been used to uncover UCHL1 as a key regulator in osteoclastogenesis^96^. UCHL1 regulates the anti-osteoclastogenic function of NRF2 by stabilizing KEAP1, thereby presenting a potential therapeutic target for bone disorders driven by excessive osteoclast activity^96,97^. A recent study identified an alternative osteoclast differentiation pathway involving CD11c^+^ dendritic cell-derived precursors (mDDOCp), which play a significant role in inflammatory bone loss^98^. These precursors, distinct from classical monocyte/macrophage-derived osteoclast progenitors, differentiate into functional osteoclasts under the influence of the pro-inflammatory cytokine interleukin-17 (IL-17) and regulatory cytokine TGF-β. Unlike traditional osteoclastogenesis, which depends on RANKL-RANK signaling, this pathway operates independently, leveraging alternative signaling cascades such as NF-κB and MAPK^98^. IL-17 enhances the osteoclastogenic potential of mDDOCp, while TGF-β synergistically promotes their differentiation and modulates the inflammatory milieu. These precursors were localized in arthritic joints, contributing to bone erosion through direct differentiation into TRAP-positive osteoclasts. This mechanism underscores the versatility of osteoclast progenitors and their adaptability under inflammatory conditions. The pathway demonstrates how osteoimmune interactions facilitate bone loss, particularly in arthritis models^98^. Understanding this alternative route highlights the importance of targeting IL-17 and TGF-β signaling as potential therapeutic strategies, not only broadening the scope of osteoclast biology but also offering new avenues for mitigating pathological bone resorption in inflammatory diseases^98^.

#### BMP signaling

**Fig. 3:**
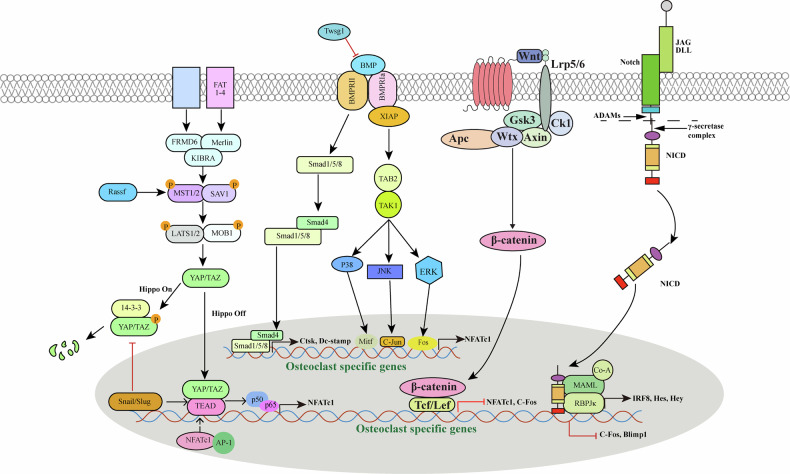
Noncanonical signaling pathways in osteoclast differentiation. This figure illustrates the complex interactions among the Wnt, Hippo, and Notch signaling pathways, emphasizing their roles in osteoclastogenesis. The binding of Wnt to the Lrp5/6 and Frizzled receptors leads to the inhibition of Gsk3β, allowing β-catenin to translocate into the nucleus. In the nucleus, β-catenin associates with Tcf/Lef transcription factors to inhibit osteoclast-specific genes like *NFATc1* and *c*-*Fos*. The Hippo pathway components MST1/2 and LATS1/2 inhibit the transcriptional co-activators YAP/TAZ by promoting their phosphorylation, leading to their cytoplasmic retention. When Hippo signaling is off, dephosphorylated YAP/TAZ translocates into the nucleus and interacts with TEAD to promote the expression of genes involved in cell proliferation and survival, which also plays roles in osteoclastogenesis. Activation of Notch by its ligands results in the cleavage of the Notch intracellular domain (NICD), which translocates to the nucleus and forms a complex with the transcription factor RBPJ to regulate the expression of genes that inhibit osteoclastogenesis.

BMP signaling regulates osteoclast differentiation through both canonical and noncanonical mechanisms, with the canonical pathway corresponding to the SMAD signaling cascade (Fig. 3), involves receptor-regulated SMADs (SMAD1/5/8) together with the common mediator SMAD4. Upon activation, R-SMADs form complexes with SMAD4 and translocate into the nucleus to regulate target gene transcription^99^. Several studies have determined that osteoclasts express SMAD and phosphorous-active SMAD^99^. Previous studies suggest that BMP-mediated SMAD signaling contributes to osteoclast fusion and activation, as SMAD inhibition yields smaller, less active osteoclasts. However, SMAD signaling remains intact in BMPRII-deficient osteoclasts, indicating that BMP signaling in these cells predominantly proceeds via noncanonical pathways^100^. This noncanonical pathway involves MAPK cascades, including JNK, p38α, and ERK, which are activated by BMP2 in osteoclasts (Fig. 3)^100^. TAK1 functions upstream in this pathway and is indispensable for osteoclast differentiation, as osteoclast-specific deletion of *Tak1* results in an osteoporotic phenotype with impaired resorptive activity^100^. Although X-linked inhibitor of apoptosis protein (XIAP) is not expressed in osteoclasts, its interaction with BMPRIa and the TAK1-binding protein 1 (TAB1) suggests additional layers of regulation in BMP-mediated signaling^100^. TAB1 associates with TAK1-binding protein 2 (TAB2) to facilitate the formation of TAK1-associated and TAK1-activated signaling complexes^100^. These findings indicate that BMP can activate noncanonical pathways in osteoclasts through the BMPR-XIAP-TAB1/TAB2-TAK1 axis, thereby stimulating MAPK signaling components such as JNK, p38, and ERK (Fig. 3)^100^. This mechanism partially overlaps with RANK-mediated signaling, which also engages the MAPK cascade but relies on TRAF6, rather than XIAP, to recruit TAB1/TAB2 and activate TAK1^100^. The convergence of BMP- and RANK-induced MAPK signaling enhances NFATc1 expression and nuclear translocation, a key event driving transcription of osteoclast-specific genes required for precursor fusion and functional activation^99^. Such overlap may potentiate osteoclastogenesis and indirectly influence bone remodeling. In addition, recent studies have examined the role of the glucose-dependent insulinotropic polypeptide analog (D-Ala²) GIP in modulating inflammatory bone resorption^101^. The compound inhibits osteoclast formation by reducing RANKL and TNF-α expression in macrophages and directly suppressing osteoclastogenesis through the MAPK pathway^101^. These findings suggest there may be some utility of GIP analogs in the therapeutic intervention in inflammatory bone diseases.

The fusion genes DC-STAMP and ATP6v0d2 are transcriptionally regulated by NFATc1, activated downstream of both RANKL and BMP signaling^102^. Inhibition of BMP reduces the expression of DC-STAMP, ATP6v0d2, and NFATc1, resulting in fewer and smaller multinucleated osteoclasts, thereby highlighting the essential role of BMP signaling in pre-osteoclast fusion^103^. Furthermore, immature osteoclasts require BMP2 to facilitate NFATc1 nuclear translocation, which in turn induces the transcription of osteoclast markers such as TRAP and CTSK (Fig. 3)^103^. Although BMP has been considered primarily as an enhancer of RANKL-mediated osteoclastogenesis, evidence from soluble BMPRIa experiments in bone marrow macrophages demonstrates that RANKL alone is insufficient to drive osteoclast differentiation in the absence of BMP signaling^102^. Collectively, these findings indicate that BMP signaling is indispensable for effective RANKL-dependent osteoclast formation.

Bone morphogenetic protein 3b (BMP3b), a non-osteogenic BMP, regulates bone mass primarily by acting as an antagonist of BMP signaling^104^. BMP3b knockout mice exhibit a high bone mass phenotype, with increased osteoblast differentiation capacity in bone marrow stromal cells, while osteoclast-related gene expression remains unchanged. Mechanistic studies revealed that BMP3b suppresses BMP4-induced Smad1/5 phosphorylation and inhibits BMP4-driven Id-1 luciferase activity by competitively interfering with BMP4–BMP type I receptor interactions^104^. These findings suggest that BMP3b functions as a BMP receptor antagonist in regulating bone mass and osteoclast differentiation, providing insights into novel therapeutic strategies for osteoporosis and bone metabolism disorders^104^.

#### Notch signaling

Notch signaling exerts a direct regulatory effect on osteoclast differentiation. Engagement of Notch ligands suppresses CSF-1R expression in OCPs, thereby limiting their ability to differentiate into mature osteoclasts (Fig. 3)^105^. Consistently, conditional deletion of Notch1–3 in macrophages enhances their osteoclastogenic potential under low concentrations of RANKL and increases their proliferative response to M-CSF relative to wild-type counterparts^106^. Moreover, RANKL stimulation induces Notch2 expression in macrophages, which is recruited to the *NFATc1* promoter through interaction with RelA, thereby facilitating NFATc1 transcription and promoting osteoclastogenesis. Canalis et al.^107^ described how NOTCH2 signaling promotes osteoclastogenesis, but inhibition of this pathway, particularly through target genes like Hes1, has been shown to reduce osteoclast activity. The deletion of Hes1 in osteoclasts led to decreased bone resorption, showcasing the importance of regulatory pathways in osteoclast behavior. Notch ligands can also suppress osteoclastogenesis indirectly by downregulating M-CSF and RANKL expression in stromal cells^106^. Consistent with this, selective activation of canonical Notch signaling in bone cells has been shown to increase bone mass in mice, accompanied by reduced osteoclast formation and bone resorption, primarily due to the downregulation of RANKL expression^106^. Conversely, osteoblast-specific deletion of Notch led to age-associated bone loss, attributable to diminished OPG production and enhanced osteoclast activity^106^. These findings suggest that Notch signaling inhibits osteoclast differentiation through dual mechanisms: directly by suppressing CSF-1R expression in OCPs, and indirectly by regulating the RANKL/OPG ratio in osteoblast-lineage cells (Figs. 1 and 5)^106^.

TNF-α predominantly enhances osteoclastogenesis and bone resorption under pathological conditions, raising questions regarding the involvement of Notch signaling in this process^108^. Recent evidence identifies RBP-J as a critical negative regulator that restrains osteoclast differentiation and limits excessive bone resorption in TNF-driven inflammatory settings^82^. Loss of RBP-J permits TNF-α to induce robust osteoclastogenesis and bone resorption even in Rank-deficient mice, whereas OCP-specific activation of RBP-J suppresses osteoclast differentiation and inflammatory bone loss in TNF transgenic models^82^. Mechanistically, RBP-J impedes NFATc1 induction by attenuating c-Fos activation and suppressing B-lymphocyte-induced mature protein-1 (Blimp1) expression, thereby maintaining transcriptional repressors such as IRF-8 that block osteoclastogenesis (Table 1)^82^. Notably, similar to p100 deletion, RBP-J deficiency does not alter the skeletal phenotype of *RANK*^*–/–*^ mice, indicating that its regulatory role is context-dependent and has limited impact on physiological bone remodeling^82^.

Decreased OPG levels have been shown to increase osteoclast generation, suggesting that Notch signaling can regulate the formation of mature osteoclasts in a non-cellular autonomous manner by downregulating OPG expression (Table 2)^109^. However, in vitro studies have also shown that Notch1 signaling can increase osteoclast production and can also be positively regulated by Notch2 during cellular autonomy^110^. Inhibit Notch1 signaling in OCPs promotes their differentiation into mature osteoclasts^105^. In contrast, Notch signaling blockade by γ-secretase inhibitors or Notch2 shRNA suppresses RANKL-induced osteoblast formation, whereas ectopic intracellular expression of Notch2 enhances osteogenesis^111^. Therefore, both cellular and non-cellular processes may be involved in the notch-dependent regulation of osteoclast differentiation. In addition, OCPs preferentially express Notch2 and express very low levels of Notch1. The general effect of Notch signaling on osteoclast generation appears to be positive (Fig. 3)^82^.

**Table 2:** Human osteoporosis- and osteopetrosis-associated genes: key genes involved in osteoclast differentiation and function defects.

Gene	Protein product	Mutation type	Disorders	Functional defects	References
ATP6i (TCIRG1)	a3 subunit of vacuolar H^+^-ATPase	Loss-of-function mutations	Autosomal recessive osteopetrosis	Defects in osteoclast proton pump activity	^400^
CAII	Carbonic anhydrase II	Loss-of-function mutations	Osteopetrosis with renal tubular acidosis	Impaired acidification	^401–403^
CLCN7	Chloride channel 7	Missense and nonsense mutations	Autosomal dominant osteopetrosis type II (ADO II)	Impaired acidification and bone resorption due to the dysfunction of chloride channel	^404–408^
CYP27B1	1α-hydroxylase	Loss-of-function mutations	Vitamin D-dependent rickets type 1 (VDDR1A)	Impaired osteoclastogenesis due to reduced RANKL and calcium availability	^409^
ESR1/ESR2	Estrogen receptor alpha and beta (ERα/β)	single-nucleotide polymorphisms (SNPs) and loss-of-function mutations	Postmenopausal osteoporosis	Loss of estrogen signaling leads to increased osteoclast activity and bone loss	^410^
FGF23	Fibroblast growth factor 23	Gain-of-function mutations	Autosomal dominant hypophosphatemic rickets (ADHR)	Defects in phosphate regulation and bone mineralization	^411^
OSTM1	Osteopetrosis-associated transmembrane protein 1	Loss-of-function mutations	Autosomal recessive osteopetrosis	Defects in osteoclast function	^412^
RANK (TNFRSF11A)	Receptor activator of nuclear factor κB	Gain-of-function mutations	Familial expansile osteolysis	Increased osteoclast formation and activity	^413^
RANKL (TNFSF11)	RANK ligand	Loss-of-function mutations	Osteoporosis	Defects in osteoclastogenesis and bone resorption	^414^
SNX10	Sorting nexin 10	Loss-of-function mutations	Autosomal recessive osteopetrosis	Impaired vesicular trafficking and osteoclast resorption activity	^415^
TNFRSF11B (OPG)	Osteoprotegerin	Loss-of-function mutations	Juvenile Paget’s disease	Increased osteoclast formation and activity	^416^
PLEKHM1	Pleckstrin homology domain-containing protein	Loss-of-function mutations	Intermediate autosomal recessive osteopetrosis	Impaired vesicular trafficking in osteoclasts	^417^
VDR	Vitamin D receptor	SNPs	Osteoporosis	Reduced receptor activity affecting calcium metabolism and osteoclastogenesis	^418^

NOTCH2 can enhance osteoclastogenesis, an effect that is mostly mediated by its target gene *Hes1*. Recent research found that Notch2^tm1.1Ecan^ mice are osteopenic and have enhanced osteoclastogenesis. Bulk RNA-Seq and gene set enrichment analysis of Notch2^tm1.1Ecan^ BMDMs cultured in the presence of M-CSF and RANKL revealed enrichment of genes associated with enhanced cell metabolism, aerobic respiration, and mitochondrial function, all of which are critical for driving osteoclastogenesis. However, these pathways were not enhanced in the context of a Hes1 inactivation^107^.

#### Hippo signaling

Components of the Hippo signaling pathway contribute to the regulation of OCP proliferation, differentiation, and distinct stages of apoptosis and survival. Upon activation, MST and LATS kinases regulate the transcriptional co-activators YAP and TAZ, which interact with TEAD family transcription factors to drive the expression of downstream target genes, including connective tissue growth factor (CTGF/CCN2) and cysteine-rich protein 61 (CYR61/CCN1), thereby modulating osteoclast differentiation^112^. Furthermore, components of the Hippo signaling pathway, including the YAP/TAZ/TEAD complex, RASSF2, MST2, and AJUBA, which may influence osteoclastogenesis by intersecting with RANKL-driven cascades such as NF-κB, MAPK, AP-1, and NFATc1 pathways (Fig. 3)^112^.

The cAMP response element-binding protein (CREB) promotes osteoclast differentiation by activating downstream transcription factors, including AP-1 and NFATc1. CREB directly promotes YAP transcription by binding to a regulatory region between nucleotide positions –608 and –439 within the *YAP* promoter. In turn, YAP stabilizes CREB through interaction with p38. Notably, phosphorylation of CREB at Ser133 by p38 facilitates CREB degradation, whereas YAP can attenuate p38 phosphorylation, as observed in cancer cells. Moreover, in hepatocellular carcinoma, YAP enhances CREB activity through AP-1 components c-Jun and c-Fos, suggesting a reciprocal regulatory loop between CREB and YAP^112^.

When transferred to the nucleus, YAP induces the expression of CTGF, which controls osteoclast formation^112^. Downregulation of the YAP target gene *CTGF* impairs OCP differentiation, whereas supplementation with recombinant CTGF restores osteoclastogenesis^112^. Mechanistically, CTGF binds RANK and amplifies RANK-induced signaling cascades, including NF-κB, p38, and JNK^112^. CTGF also interacts with OPG, the decoy receptor for RANKL, thereby modulating ligand–receptor availability. In vivo, CTGF expression is upregulated by day 6 following RANKL stimulation, and combined treatment with CTGF and RANKL markedly enhances the formation of TRAP-positive multinucleated cells^112^. Conversely, another YAP/TAZ downstream target, CYR61/CCN1, serves as a negative regulator of osteoclastogenesis. CYR61 inhibits the formation of TRAP-positive multinucleated cells and downregulates osteoclastic marker expression, exerting its effects primarily on early OCPs rather than on mature osteoclasts^112^. Importantly, CYR61 does not affect RANKL-induced phosphorylation of p38, ERK1/2, or NF-κB, indicating that its inhibitory action occurs via a RANK-independent mechanism that has yet to be fully clarified (Fig. 2)^112^.

#### RGS family and RANK signaling

**Fig. 4:**
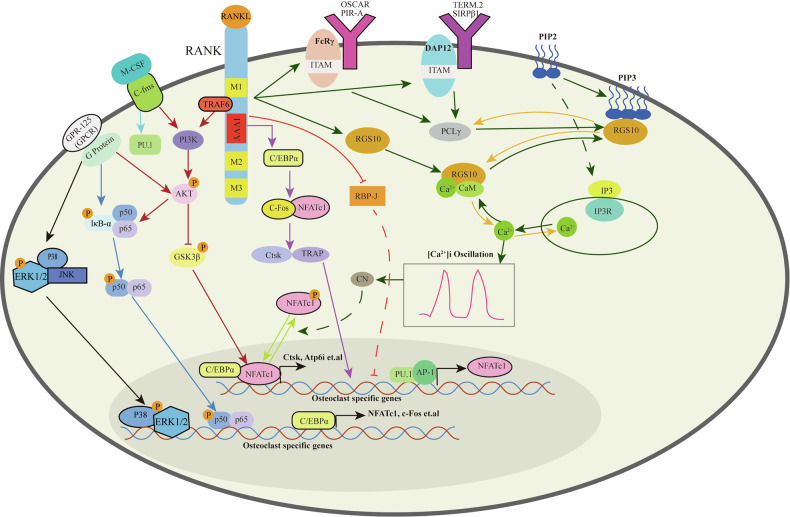
Signaling and transcriptional regulation of osteoclast cell lineage commitment, differentiation. This figure illustrates the complex network of intracellular signaling pathways that regulate osteoclast differentiation and function, highlighting the roles of key receptors, transcription factors, and second messengers in the process. RANKL binding to RANK activates a cascade of intracellular signaling events, beginning with the recruitment of TRAF6, which plays a pivotal role in downstream signal transduction. Additionally, PU.1 is involved in the activation of C/EBPβ, further enhancing NFATc1 and c-Fos activity. The activation of AKT leads to the inhibition of GSK3β, allowing for the accumulation of β-catenin, which synergizes with NFATc1 to promote osteoclastogenesis. OSCAR, PIR-A, TREM-2, and SIRPβ1 receptors initiate co-stimulatory signals via DAP12 and FcRγ, which are crucial for full osteoclast differentiation. These receptors activate ITAM signaling, leading to the recruitment and activation of PLCγ. PLCγ activation results in the production of IP3, which induces the release of calcium from intracellular stores, generating [Ca^2+^]_i_ oscillations. These calcium oscillations are essential for the activation of NFATc1, driving its nuclear translocation and promoting osteoclast-specific gene expression. RGS10 modulates signaling pathways by interacting with CaM and the Gα_i_ subunit, fine-tuning calcium signaling and calcium oscillations. RGS12 also plays a significant role in RANKL-evoked signaling by controlling the terminal differentiation of osteoclasts, linking G protein-coupled signaling with osteoclastogenesis. The MAPK pathway involves the activation of ERK1/2 and p38, which phosphorylate downstream targets, including c-Fos and NF-κB components, contributing to the transcriptional regulation of osteoclast genes. NF-κB activation occurs through the degradation of IκBα, releasing p50/p65 to translocate into the nucleus and synergize with NFATc1 in promoting osteoclast differentiation. The figure integrates multiple signaling pathways that converge on the activation of NFATc1 and other transcription factors, which are critical for osteoclast differentiation and function. Crosstalk between these pathways ensures the precise regulation of osteoclast-specific gene expression, ultimately controlling the formation, activation, and survival of osteoclasts.

Regulators of G protein signaling (RGS) function as GTPase-activating proteins (GAPs) for Gα subunits, thereby terminating G protein-mediated signaling responses in eukaryotic cells^113–115^. The occupation of the receptor with an agonist initiates the cycle of inhibition of RGS protein action by binding to phosphatidylinositol (3,4,5)-trisphosphate (PIP3) and alleviates the inhibition by binding to Ca^2+^/calmodulin (CaM) to promote cyclic activation of PLC, intermuscular 1, 5-triphosphate (IP3) production, and Ca^2+^ release. RGS periodically regulates Ca^2+^ signaling, resulting in intracellular Ca^2+^ concentration ([Ca^2+^]_i_) oscillations (Fig. 4)^116,117^.

RGS12, the largest member of the RGS protein family, acts as a central signaling hub that integrates and modulates multiple cellular pathways through its multidomain structure^118^. It contains an RGS domain, a GoLoco motif, two Ras-binding domains (RBDs), multiple N-terminal PSD-95/Dlg/ZO-1 (PDZ) domains, and a phosphotyrosine-binding (PTB) domain^118^. RGS12 plays an important role in Rank-induced osteoclast terminal differentiation as a key regulator of Ca^2+^ oscillation^119,120^. Knockdown of RGS12 enhances PLCγ phosphorylation, disrupts intracellular Ca^2+^ oscillations, and reduces NFAT2 expression and osteoclast differentiation, suggesting that RGS12 modulates PLCγ activation and Ca^2+^ signaling via the GTPase-activating function of its RGS domain^121^. The initiation and maintenance of Ca^2+^ oscillations require both Ca^2+^ release from endoplasmic reticulum (ER) stores and extracellular Ca^2+^ influx through multiple channels, including L- and N-type channels (Fig. 4)^121^. Consistently, our previous work showed that RANKL-induced osteoclasts exhibit increased RGS12 expression and elevated tyrosine phosphorylation of N-type calcium channels, with RGS12 directly interacting with the phosphorylated channels (Table 1)^121^. Furthermore, RGS12 expression peaks within the first 2 h following RANKL stimulation, whereas intracellular Ca^2+^ oscillations become evident between 24 h and 72 h post-induction^121^. Notably, silencing RGS12 inhibited both [Ca^2+^]_i_ oscillations and osteoclast differentiation^121^. These findings suggest that RANKL induces RGS12 expression before the initiation of Ca^2+^ oscillations. Through interaction with N-type calcium channels, RGS12 modulates transient intracellular Ca^2+^ fluxes, thereby generating oscillations that promote osteoclast differentiation^121^. In addition to regulating the expression of NFATc1, RGS12 can also regulate the expression of c-Fos (Fig. 4). LPS enhances the regulatory role of RGS12 in osteoclast differentiation by activating toll-like receptor 2 (TLR2) and toll-like receptor 4 (TLR4) in pre-osteoclasts or OCPs, thereby promoting their differentiation^122,123^.

In addition to RGS12, RGS10 is also expressed in osteoclasts and plays a positive role in regulating osteoclast differentiation (Fig. 4). The A isoform of RGS10, RGS10A, plays a major role in the terminal differentiation of osteoclasts^124^. RANKL induces phosphorylation of FcRγ and DAP12, immunoreceptor tyrosine-based activation motif (ITAM)-containing adaptor molecules, thereby activating PLCγ^125^. Activated PLCγ hydrolyzes phosphatidylinositol 4,5-bisphosphate (PtdIns(4,5)P₂) to generate inositol 1,4,5-trisphosphate (Ins(1,4,5)P₃), which triggers an initial transient release of Ca^2+^ from intracellular stores^125^. The release of intracellular Ca^2+^ elevates cytosolic Ca^2+^ to peak levels, promoting the formation of Ca^2+^/CaM complexes^125^. Ca^2+^/CaM complexes compete with RGS10A for binding to phosphatidylinositol (3,4,5)-trisphosphate (PtdIns(3,4,5)P₃) sites, thereby displacing PtdIns(3,4,5)P₃ from RGS10A (Fig. 4)^125^. Once [Ca^2+^]ᵢ reaches its peak, Ca^2+^ is subsequently re-sequestered into the ER through mechanisms independent of further PLCγ activity. Binding of Ca^2+^ to the ER and to CaM reduces cytosolic Ca^2+^ levels^125^. The Ca^2+^/CaM complex dissociates from RGS10A at low Ca^2+^ concentration^125^. Free phosphatidylinositol (3,4,5)-trisphosphate (PtdIns(3,4,5)P₃) activates PLCγ and, once Ca^2+^/CaM complexes dissociate, rebinds to RGS10A in the absence of competitive inhibition^125^. PLCγ activation generates Ins(1,4,5)P₃, which releases Ca^2+^ from intracellular stores and produces a secondary Ca^2+^ spike^125^. This cycle continues, causing [Ca^2+^]_i_ oscillations^125^. Thus, RGS10A mediates PLCγ activation and induces [Ca^2+^]ᵢ oscillations through its Ca^2+^-dependent dual interactions with Ca^2+^/CaM and PtdIns(3,4,5)P₃^125^. RGS10A-mediated [Ca^2+^]ᵢ oscillations activate calcitonin and NFAT2 expression, driving terminal osteoclast differentiation^125^. RGS12 possesses multiple interaction domains, including RBD, PDZ, and PTB, as well as an uncharacterized GoLoco motif, whereas RGS10A contains only the canonical RGS domain. Notably, the RGS domain of RGS12 shares only 51% sequence similarity with that of RGS10A, indicating structural divergence. These differences suggest that RGS12 and RGS10A may engage distinct molecular targets, thereby conferring specificity in the regulation of intracellular Ca^2+^ oscillations (Fig. 4)^121^.

#### Src kinase positively regulates osteoclast differentiation

Among the intracellular kinases that regulate osteoclast differentiation, Src kinase represents one of the most critical positive regulators linking extracellular cues to cytoskeletal and functional activation^126^. Src is a non-receptor tyrosine kinase abundantly expressed in osteoclasts, where it mediates key signaling events downstream of RANK–RANKL interactions — the canonical pathway that governs osteoclast differentiation and function^127^. Upon RANKL stimulation, RANK recruits adaptor proteins such as TRAF6, which subsequently activate c-Src and phosphatidylinositol 3-kinase (PI3K) signaling pathways, initiating downstream cascades essential for osteoclast activation and function^127^.

Activated Src phosphorylates multiple target proteins, including p130Cas, Pyk2, c-Cbl, and Vav3^128^, which coordinate cytoskeletal reorganization and facilitate actin ring formation, a hallmark structure of mature, resorptive osteoclasts^129^. These adaptor-mediated interactions promote the development of the ruffled border, a specialized membrane domain critical for bone matrix degradation^129^. Through these actions, Src ensures the mechanical and functional competence of osteoclasts. In addition to its cytoskeletal role, Src indirectly influences transcriptional activation^126^. It facilitates the induction of NFATc1, the master transcription factor governing osteoclast differentiation, through activation of the PLCγ-Ca^2+^-calcineurin signaling cascade^130^. This signaling cascade upregulates the expression of osteoclast-specific genes, including CTSK, TRAP, and DC-STAMP, which are essential for effective bone resorption^131^.

The physiological importance of Src in osteoclast biology is strongly supported by genetic evidence. Mice lacking Src (*Src*^*−*^^/^^*−*^) develop osteopetrosis, a disorder characterized by abnormally high bone mass resulting from defective bone resorption^132^. Notably, these mice generate morphologically normal osteoclasts that fail to resorb bone, reflecting disrupted cytoskeletal architecture and absence of ruffled borders. This phenotype indicates that Src is not essential for osteoclast formation itself but is crucial for their activation and functional maturation^132^. These findings establish Src kinase as a pivotal positive regulator in osteoclast differentiation and function^132^. Through its dual role in cytoskeletal organization and transcriptional activation, Src integrates RANKL signaling into the structural and enzymatic machinery that drives bone resorption. Dysregulation or inhibition of Src activity, therefore, profoundly impairs osteoclast-mediated bone remodeling and contributes to osteopetrotic phenotypes^132^.

In addition to these classical pathways, cytokines that positively regulate osteoclast differentiation are further discussed in “Cytokine and growth factors regulating osteoclast differentiation and activation” section, emphasizing the multifactorial nature of osteoclastogenic signaling.

### Negative regulation of osteoclastogenesis

#### Gα_13_ and RANKL pathway in osteoclast suppression

Emerging research highlights the pivotal roles of Gα_13_ and the RANKL signaling pathway in suppressing osteoclast differentiation and activity, offering potential therapeutic avenues for conditions associated with excessive bone resorption. Previous studies demonstrate that Gα_13_ suppresses osteoclast differentiation by inhibiting the activation of NFATc1, a master transcription factor essential for this process (Table 1)^133^. Similarly, Zhu et al.^134^ demonstrated that Puerarin disrupts osteoclast activation by targeting the integrin-β3 Pyk2/Src/Cbl signaling pathway, revealing another mechanism to inhibit bone resorption. Consistent with this, Teitelbaum and colleagues identified Src kinase as a critical regulator of osteoclast differentiation, and its inhibition markedly diminishes osteoclast activity^135^. Zheng et al.^136^ further substantiated this by showing that Src siRNA markedly decreases osteoclast activity, presenting a promising strategy to combat corticosteroid-induced bone loss.

Complementary findings by Bin Karim et al. underscore the importance of modulating the RANK/RANKL/OPG axis in osteoclast suppression^137^. Their work with *Marantodes pumilum* extract in a postmenopausal rat model revealed that increasing OPG levels and decreasing RANKL effectively suppresses osteoclast activation and prevents bone loss^137^. Collectively, these studies highlight the interconnected pathways regulating osteoclast function and provide valuable insights into strategies for mitigating osteoclast-mediated bone disorders.

#### Upstream and downstream regulation of IRF8 in osteoclast differentiation

IRF8 serves as a master transcriptional repressor of osteoclast differentiation and a key molecular checkpoint that maintains skeletal homeostasis^138^. Highly expressed in myeloid progenitor cells and early OCPs, IRF8 acts to restrain the activation of the RANKL-NFATc1 signaling axis and preserve the undifferentiated state of OCPs^8^. Mechanistically, IRF8 directly binds to the promoter region of NFATc1, competing with transcriptional activators such as NF-κB and c-Fos for promoter occupancy and preventing the recruitment of co-activators required for transcriptional activation^139^. Through this direct repression, IRF8 inhibits the induction of osteoclast-specific genes including *CTSK*, acid phosphatase 5 (*Acp5*), *Dcstamp*, and osteoclast-associated receptor (*Oscar*), thereby suppressing both differentiation and bone-resorptive function^139^. In addition to this promoter-level regulation, IRF8 forms inhibitory complexes with PU.1 and BCL6, establishing a repressive transcriptional network that stabilizes precursor identity^140^. These actions collectively function as a transcriptional barrier preventing premature osteoclastogenesis under physiological conditions. Genetic studies further demonstrate that IRF8-deficient mice (*IRF8*^*−*^^/^^*−*^) display severe osteopenia accompanied by excessive osteoclast formation and heightened bone resorption, underscoring its indispensable role in restraining osteoclast differentiation^138^.

IRF8 expression and activity are dynamically regulated during osteoclastogenesis. Upon RANKL stimulation, the transcriptional repressor Blimp1 (PRDM1) is induced downstream of NF-κB and AP-1 pathways^141^. Blimp1 directly binds to the *IRF8* promoter and suppresses its transcription, establishing the Blimp1-IRF8 regulatory axis as a pivotal molecular switch controlling the transition from inhibition to activation of osteoclastogenesis^141^. RANKL signaling induces post-translational downregulation of IRF8 through ubiquitin–proteasome-mediated degradation driven by the E3 ubiquitin ligase Cbl-b, thereby facilitating efficient activation of NFATc1^142^. These multilevel regulatory mechanisms ensure the precise and timely removal of IRF8-mediated repression during differentiation, enabling the full activation of the osteoclast transcriptional program.

Beyond its transcriptional repression of NFATc1, IRF8 exerts broad influence across multiple osteoclastogenic signaling pathways. IRF8 interferes with RANKL-induced NF-κB and MAPK (ERK, JNK, p38) activation, attenuating downstream c-Fos induction and preventing the feed-forward amplification of NFATc1^143^. It also associates with other members of the interferon (IFN) regulatory factor family, such as IRF1 and IRF5, to regulate inflammatory gene expression in OCPs. Importantly, IRF8 expression is sustained or upregulated by anti-osteoclastogenic cytokines such as interferon-γ (IFN-γ) and interleukin-27 (IL-27). Both cytokines act through the STAT1 signaling pathway to maintain IRF8 levels and suppress the RANK-NFATc1 axis^143^. Thus, IRF8 functions as a molecular node integrating immune-derived inhibitory signals with intrinsic transcriptional control, forming a bridge between osteoimmunology and bone remodeling.

The biological relevance of IRF8 extends beyond cellular differentiation to organismal bone homeostasis. Cytokine-mediated induction of IRF8, particularly by IFN-γ and IL-27, protects against excessive osteoclast activation and inflammatory bone destruction in arthritis and periodontitis models^8^. This reciprocal balance defines a feedback circuit in which RANKL-induced Blimp1 suppresses IRF8 to enable osteoclast differentiation, while immune cytokines restore IRF8 expression to restrain pathological bone resorption^8^. Through this dynamic regulation, IRF8 functions as both a transcriptional brake and a signaling integrator, ensuring that osteoclast formation remains precisely coupled to physiological remodeling demands.

#### Estrogen and osteoclast apoptosis

A study found that estrogen prevents osteoclast activity by inducing apoptosis through the upregulation of the Fas ligand, which limits osteoclast lifespan^144^. Zhang et al.^145^ added to this by showing that estrogen receptor-beta influences osteoclast activity during fracture healing, suggesting estrogenic modulation of osteoclasts in bone repair (Tables 1 and 2). These studies highlight various mechanisms through which osteoclast activity is negatively regulated, including the Gα_13_ pathway, Src kinase inhibition, and hormonal regulation via estrogen. Understanding these negative regulatory mechanisms opens the door to new therapeutic approaches for diseases characterized by excessive bone resorption.

In addition, cytokines that negatively regulate osteoclast differentiation are discussed in “Cytokine and growth factors regulating osteoclast differentiation and activation” section, further illustrating the multifaceted control of osteoclastogenesis.

## Regulation of transcription factors during osteoclast differentiation

Eukaryotic gene transcription is controlled by DNA-binding transcription factors that assemble on *cis*-regulatory elements located within promoters and enhancers. While many of these transcription factors are ubiquitously expressed and participate in general transcriptional regulation, others display lineage-restricted expression patterns. Such cell type-specific transcription factors act as key regulators of lineage-defining genes in skeletal muscle, neuronal, erythroid, myeloid, and lymphoid lineages^146–150^.

Gene-targeting studies have revealed that distinct transcription factors control osteoclast differentiation, activation, and survival, with several functioning downstream of M-CSF and RANKL/RANK signaling. For example, macrophage development remains intact in NF-κB-deficient mice, suggesting that NF-κB operates at a later stage of osteoclastogenesis than PU.1 (Fig. 4)^151,152^. Loss of c-Fos results in severe osteopetrosis due to a complete absence of osteoclasts (Table 1)^151,152^. NFATc1 serves as a master regulator of terminal osteoclast differentiation downstream of RANKL signaling (Fig. 2)^143^. In addition, the microphthalmia-associated transcription factor (MITF) and related members of the MiT/TFE family (TFE3, TFEB, and TFEC) are essential for the fusion of mononuclear precursors into multinucleated osteoclasts^153^. These transcription factors link RANK-mediated signaling to the activation of osteoclast-specific genes, including calcitonin receptor (CTR), TRAP, CTSK, osteoclast-associated receptor, and αvβ3 integrins (Fig. 2).

### PU.1

PU.1, a member of the ETS family of transcription factors, is critical for macrophage and osteoclast development by actively controlling the expression of c-Fos (Fig. 4).

PU.1 plays a central role in lymphoid and myeloid lineage development by regulating cytokine receptor genes such as CSF1R, GM-CSFRα, G-CSFR, and IL-7Rα^154^. PU.1 is crucial for macrophage development during the transition from pro-monocytes to monocytes and remains expressed throughout osteoclast differentiation, with its mRNA levels rising by roughly 3-fold as cells mature^155^. PU.1-deficient mice exhibit osteopetrosis and a complete absence of both osteoclasts and macrophages, indicating a differentiation arrest at the M-CSF-dependent precursor stage (Table 1)^155^. Mechanistically, PU.1 binds directly to promoters and enhancers of osteoclast-specific genes. Notably, RANK, a central regulator of osteoclastogenesis, is a direct transcriptional target of PU.1. In PU.1-deficient progenitors, RANK expression is absent but can be restored following PU.1 reintroduction^11,156^. Moreover, PU.1 cooperates with NFATc1 to regulate RANKL-induced CTSK transcription^157^ and with MITF to activate TRAP expression via the *TRAP* gene promoter (Fig. 4)^158^. Collectively, these findings identify PU.1 as a master regulator that governs the development of common progenitors in lymphoid-myeloid lineages and orchestrates osteoclast-specific gene expression during osteoclastogenesis. C/EBP-α is the target gene of PU.1. PU.1 can be induced by the key IVVY motif of RANK, but this is not necessary for PU.1 expression^11^. The fact that RANK IVVY motifs inactivation inhibited C/EBP-α during osteoclast differentiation despite normal PU.1 expression suggests that PU.1 may interact with other unknown factors regulated by RANK IVVY motifs to upregulate C/EBP-α during osteoclast differentiation (Fig. 4)^11^.

During osteoclast differentiation, MITF and PU.1 cooperate to activate target genes such as CTSK and Acp5^159^. These factors also inhibit the transcription of target genes in osteoblast precursors that can form macrophages or osteoclasts. Direct interaction of MITF and PU.1 with zinc finger protein Eos, a member of the Ikaros family, is required to inhibit CTSK and Acp5^159^. Eos forms complexes with MITF and PU.1 at the target gene promoter and inhibits transcription by recruiting core inhibitors C-terminal binding protein (CTBP) and Sin3A, but the association of Eos with *CTSK* and *Acp5* promoters is significantly reduced during osteoclast differentiation^159^. MITF and PU.1 further recruit coactivators to their target loci, thereby enhancing transcriptional activation of osteoclast-specific genes^159^. Overexpression of Eos in bone marrow-derived precursors disrupts osteoclast differentiation by selectively repressing MITF/PU.1 targets, whereas Eos knockdown via siRNA increases basal expression of CTSK and Acp5^159^.

### AP-1

AP-1 is a dimeric transcription factor complex composed of members of the Fos (c-Fos, FosB, Fra-1, Fra-2), Jun (c-Jun, JunB, JunD), and ATF (ATFa, ATF2, ATF4, B-ATF) families^160^. Fos-related antigen 1 (Fra-1), a member of the Fos family, serves as a transcriptional target of c-Fos during osteoclastogenesis and can functionally compensate for c-Fos deficiency both in vitro and in vivo^160^. However, conditional deletion of Fra-1 does not lead to the development of osteosclerosis^160^. FosB and Fra-2 can also partially alleviate the differentiation blockade observed in c-Fos-deficient cells in vitro, although their compensatory effects are relatively modest^160^. These results suggest that c-Fos plays a dominant role in osteoclast formation in the Fos family^160^.

Disruption of the c-Fos proto-oncogene results in a severe osteopetrotic phenotype^161^. Loss of c-Fos halts osteoclast differentiation at the stage where cells diverge from the common M-CSF dependent precursor, prior to the onset of TRAP expression. Mechanistically, RANKL induces Fos-dependent transcription of downstream targets, including Fra-1 and NFATc1 (Fig. 4)^161^. Among these, NFATc1 is the pivotal effector, as ectopic expression of NFATc1 restores osteoclast formation in c-Fos-deficient bone marrow monocytes, demonstrating that NFATc1 acts as the critical downstream mediator of c-Fos in osteoclastogenesis. Additionally, it has been previously reported that NFATc1 expression cannot be activated in LIM domain-containing protein 1 (LIMD1) knockout osteoclasts, whereby LIMD1 is upregulated during this process and interacts with TRF6, positively influencing TRF6’s ability to activate AP-1, thus highlighting the critical role of LIMD1 in osteoclast differentiation^160^.

### NFATc1

Originally identified in studies of T-cell activation, NFAT transcription factors have since been recognized as key regulators of development and function across various cell types, including those in the cardiovascular and muscular systems. The NFAT family consists of five members: NFATc1 (NFAT2), NFATc2 (NFAT1), NFATc3 (NFAT4), NFATc4 (NFAT3), and NFAT5^162^. With the exception of NFAT5, which is activated by osmotic stress, all NFAT family members are mainly regulated by the Ca^2+^-dependent serine/threonine phosphatase calcineurin (Fig. 4)^162^. Dephosphorylation of serine residues in NFAs by calcineurin results in exposure of its nuclear localization signal and translocation to the nucleus. In osteoclasts, NFATc1 undergoes effective nuclear translocation in response to RANKL stimulation, suggesting activation of Ca^2+^-calcineurin signaling^162^. NFATc1 is the predominantly induced NFAT isoform in human osteoclasts, with expression levels far exceeding those of NFATc2 through NFATc4^162^. It is necessary for c-Fos to express NFATc1 since it can regulate the expression of TRAP, CTSK, matrix metalloproteinase-9 (MMP-9), and other key genes in OCPs^163,164^. NFATc1-deficient embryonic stem cells are unable to differentiate into osteoclasts in response to RANKL, whereas ectopic expression of NFATc1 induces robust osteoclast formation even without RANKL stimulation, confirming NFATc1 as the key regulator of terminal osteoclast differentiation downstream of RANKL signaling^143^. RANKL activates NFATc1 through the calcium-calcineurin signaling cascade, and pharmacological inhibitors such as FK506 or cyclosporin A markedly suppress osteoclast formation by blocking this pathway^143^. FK506-mediated inhibition impairs *NFATc1* mRNA induction, indicating an autoregulatory loop in which NFATc1 amplifies its own transcription, likely through promoter binding^165^. Moreover, NFATc1 transcription is driven by the RANKL/TRAF6/Fos pathway, as enforced NFATc1 expression restores osteoclast differentiation in c-Fos-deficient precursors^143,161^.

Sustained activation of NFATc1 mediated by Ca^2+^ signaling and cooperation with AP-1 are prerequisites for robust induction of NFATc1. Activation of AP-1 is mediated through the induction of c-Fos, which is regulated by CaM-dependent protein kinase IV (CaMKIV)-activated CREB as well as by c-Fos signaling^166^. The *NFATc1* promoter is epigenetically activated by histone acetylation, and NFATc1 binds to the NFAT-binding site on its promoter^166^. In the nucleus, NFATc1 acts together with other transcription factors such as AP-1, PU.1, CREB, and small eye-associated transcription factor (MITF) to induce various osteoclast-specific genes (Fig. 4)^166^. Calcineurin inhibitors such as FK506 and cyclosporin A, as well as the Ca^2+^ chelator BAPTA-AM, strongly inhibit RANKL-induced osteoclastogenesis by preventing NFATc1 nuclear translocation, highlighting the essential role of the Ca^2+^-NFATc1 signaling pathway in osteoclast development^166^. Moreover, forced expression of NFATc1 induces osteoclast differentiation in BMDMs even without RANKL stimulation, further confirming NFATc1 as the master regulator of osteoclastogenesis^166^.

### MITF

The basic/helix-loop-helix/leucine-zip transcription factor MITF is one of the nuclear targets of the M-CSF signaling pathway in osteoclasts. M-CSF induces MITF phosphorylation on a consensus sequence phosphorylated by MAPK^167^. EAOMES is a functional partner of PU.1 and MITF in regulating and managing osteoclast’s differentiated transcription factor networks. Previous research found that MP-specific EAOMES deletion disrupted the PU.1-MITF transcription factor network and led to osteoporosis in vivo^167^. Historically, EAES-culled mice were arrested at the blastocyst stage, resulting in early embryo death. It has been reported that Vav-Cre was used to remove blood line-specific EAOMES from the hematopoietic layer structure^167^. However, despite a global decline in NK cells from lymph, no investigation of changes in bone marrow cell lineage has been reported^167^.

MITF cooperates with PU.1 or PU.1-associated proteins to synergistically promote the expression of osteoclast-specific markers such as TRAP^158^. At the transcriptional level, MITF and TFE3 regulate CTSK expression via three consensus elements within its promoter. Similarly, PU.1 and MITF jointly activate the OSCAR gene, which encodes a bone-specific Ig-like receptor critical for osteoclast differentiation (Fig. 4)^168^. MITF is also a downstream target of RANKL signaling, where its phosphorylation enhances osteoclast-specific gene expression^169^. In addition, M-CSF can promote phosphorylation of MITF and TFE3 through a conserved MAPK consensus site, facilitating the recruitment of the coactivator p300 and further driving transcriptional activation^170^. β-glucan-induced trained immunity (TRIM) has been shown to remodel bone marrow and peripheral monocytes/OCPs, leading to an enhanced predisposition toward osteoclast differentiation^171^. This process involves the activation of critical transcription factors, including MITF, which promotes the expression of genes linked to osteoclast development in both bone marrow and synovial tissues^171^. Consequently, TRIM amplifies inflammatory osteoclastogenesis and contributes to exacerbated bone loss in experimental models of periodontitis and arthritis^171^.

### C/EBPα

C/EBPα is a transcription factor in the C/EBP family whose members share the conserved leucine zipper dimerization domain^172^. C/EBPα plays a crucial role in hematopoiesis and granulocyte formation by inducing myeloid lineage-specific genes and regulating the transition from proliferation to differentiation within the cell cycle^173,174^. Thus, C/EBPα can bind lineage commitment to terminal cell differentiation (Fig. 4)^175,176^. In addition to other homeostatic abnormalities, global deletion of the *C/EBPα* gene in *C/EBPα*^*−*^^/^^*−*^ mice results in early postnatal death and severe agranulocytosis due to a failure in granulocyte maturation^177,178^. Consistently, conditional deletion of the C/EBPα gene in adult mice impairs granulocyte differentiation and leads to an accumulation of myeloblasts^179^. C/EBPα plays an important role in osteoclast differentiation, promoting the transformation of MBM into osteoclasts, and plays a very important regulatory role in both early and late differentiation of osteoclasts, especially in the early differentiation^180^. Previous research has shown that C/EBPα deletion can lead to impaired osteoclast differentiation, showing a severe osteosclerosis phenotype, and the expression levels of key genes NFATc1, CTSK and TRAP during osteoclast differentiation are significantly decreased, suggesting that C/EBPα plays a key role in osteoclast differentiation^180^.

The C/EBPα protein is distinguished by its interaction with the promoter and contains two isomers, p42 C/EBPα and p30 C/EBPα. The p42 isoform of C/EBPα possesses two transcriptional activation domains (TAD1 and TAD2) at its N-terminus and a basic leucine zipper domain at the C-terminus, while the p30 isoform lacks the first 117 amino acids of the N-terminus, including the TAD1 region^181^. While the TAD1 and TAD2 domains are responsible for recruiting coactivators and remodeling transcriptional complexes, the basic leucine zipper domain mediates protein–protein interactions and facilitates DNA binding required for gene expression^182^. Previous studies indicate that p30 C/EBPα, lacking the TAD1 domain, can only partially support osteoclast differentiation^182^. Nevertheless, rat p30 C/EBPα retains the TAD2 domain, which, together with the basic leucine zipper region, compensates for the loss of TAD1 to promote osteoclast differentiation^182^.

C/EBPα can be activated through AKT signaling downstream of the NF-κB pathway, subsequently enhancing the expression of key osteoclastogenic genes such as NFATc1^183^, CTSK, and TRAP, thereby promoting osteoclast differentiation (Fig. 4)^180^. Overexpression of C/EBPα can initiate osteoclast differentiation independently of RANKL, and the effect of C/EBPα on osteoclast differentiation is stronger than that of c-Fos^184^. Previous work in our laboratory has shown that c-Fos is also the target gene of C/EBPα in promoting osteoclast differentiation^184^. In addition, although c-Fos gene expression was normal, the expressions of c-Fos and C/EBPα target gene NFATc1 were inhibited by the inactivation of C/EBPα due to the deletion of IVVY motifs of RANK, suggesting that C/EBPα was a more critical factor in promoting osteoclast differentiation than c-Fos^185^. C/EBPα can also promote the terminal differentiation of osteoclasts by regulating the expression of NFATc1 in the late stage of osteoclast differentiation^186^. C/EBPα can directly interact with CCER, the key *cis*-regulatory element of *NFATc1* promoter, to enhance promoter activity and promote the expression of NFATc1^186^.

### Emerging regulators

A recent study identifies sterol regulatory element-binding protein 2 (SREBP2) as a negative regulator of osteoclast formation and inflammatory bone loss, unveiling a novel feedback mechanism in bone biology^187^. SREBP2, a transcription factor involved in cholesterol biosynthesis, was shown to increase during the late stages of osteoclastogenesis. Deleting SREBP2 in myeloid cells led to heightened osteoclastogenesis both in vitro and in vivo, resulting in low bone mass and exacerbated bone destruction in murine models of inflammatory osteolysis and arthritis^187^. Importantly, this regulatory role of SREBP2 was independent of its canonical function in cholesterol biosynthesis. Instead, SREBP2 acted through its downstream target, interferon regulatory factor 7 (IRF7), forming a feedback loop to restrict osteoclast differentiation. The findings highlight the SREBP2-IRF7 axis as a critical mechanism to control osteoclastogenesis and prevent excessive bone resorption^187^. This work not only broadens our understanding of osteoclast biology but also identifies potential therapeutic targets for diseases involving pathological bone destruction^187^.

## Epigenetic regulation of transcriptional factors

Epigenetics refers to heritable changes in gene and protein expression that occur independently of alterations in the DNA sequence and are mediated through modifications in chromatin states^188^. These modifications are reversible, highly dynamic, and responsive to both endogenous signals and environmental stimuli^189^. DNA methylation, which involves the conversion of cytosine to 5-methylcytosine, predominantly occurs at CpG sites — cytosine residues located 5′ to guanosine — within CpG islands in mammals^190^. This modification can either inhibit or facilitate the binding of regulatory proteins to DNA, thereby influencing gene expression. Generally, DNA hypermethylation is associated with gene silencing. DNA methyltransferases (DNMTs) catalyze DNA methylation using S-adenosyl methionine as the methyl donor. Additionally, post-translational modifications of histone complexes, such as acetylation and methylation at specific amino acid residues on histone tails, modulate chromatin structure and histone–DNA interactions, ultimately regulating gene expression levels^191^. This section explores the epigenetic regulation of transcription factors involved in osteoclastogenesis.

### DNA methylation

**Fig. 5:**
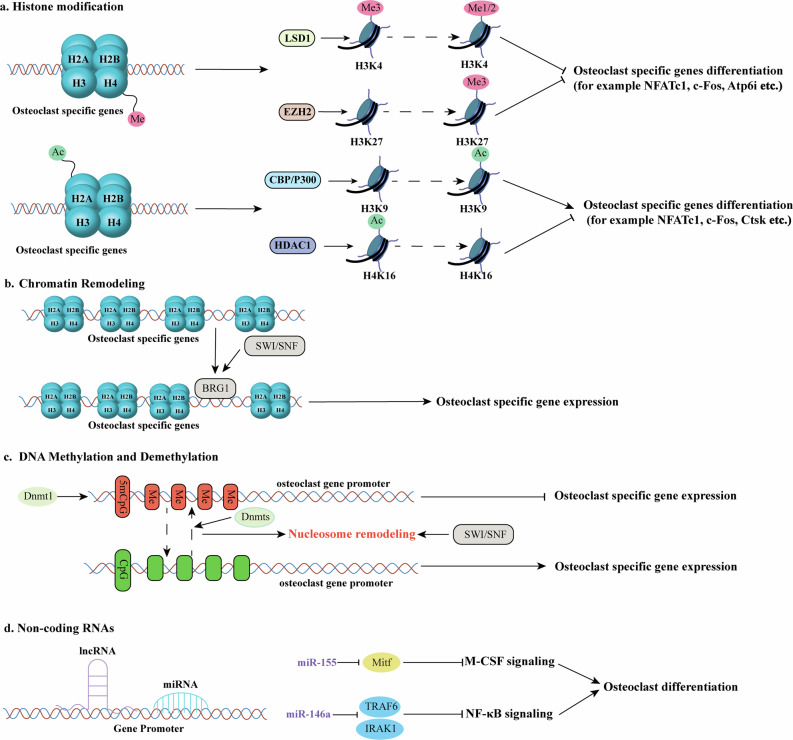
Epigenetic regulation of osteoclast differentiation. This figure provides a comprehensive overview of the various epigenetic mechanisms involved in regulating osteoclast-specific gene expression during osteoclast differentiation. The figure is divided into four main sections: histone modification, chromatin remodeling, DNA methylation and demethylation, and non-coding RNAs. **a** Histone modification: Histone modifications play a pivotal role in regulating the accessibility of osteoclast-specific genes. Key histone modification enzymes include: LSD1 (Lysine-specific demethylase 1): this enzyme demethylates H3K4me1/me2 marks, which are associated with gene activation, thus repressing osteoclast-specific gene expression. EZH2: EZH2 catalyzes the trimethylation of H3K27 (H3K27me3), a mark linked to gene repression, thereby silencing osteoclast-specific genes. CBP/p300: these HATs acetylate H3K9, a modification associated with transcriptional activation, facilitating the expression of osteoclast-specific genes like *NFATc1* and *c-Fos*. HDAC1: HDAC1 removes acetyl groups from H4K16, leading to a repressed chromatin state and decreased osteoclast-specific gene expression. **b** Chromatin remodeling: chromatin remodeling is essential for the dynamic regulation of gene expression. The SWI/SNF complex and BRG1 (Brahma-related gene 1) are key chromatin remodelers that modify the chromatin structure, making osteoclast-specific genes accessible for transcription. This process is crucial for the expression of genes necessary for osteoclast differentiation and function. **c** DNA methylation and demethylation: DNA methylation at the promoters of osteoclast-specific genes typically represses gene expression. Dnmt1 (DNA methyltransferase 1) maintains DNA methylation during cell division, while DNMT3a/b is responsible for de novo methylation. Demethylation occurs through the action of TET enzymes and is associated with the activation of osteoclast-specific gene promoters. This process is coupled with nucleosome remodeling by the SWI/SNF complex, leading to the activation of gene transcription necessary for osteoclast differentiation. **d** Non-coding RNAs: non-coding RNAs such as lncRNAs and miRNAs regulate osteoclast differentiation by modulating the expression of target genes. miR-155 targets MITF, influencing M-CSF signaling and thereby osteoclast differentiation. miR-146a regulates NF-κB signaling by targeting TRAF6 and IRAK1, both of which are crucial for osteoclastogenesis.

DNA methyltransferase 3a (DNMT3a) has been identified as a key regulator in osteoclast differentiation. Nishikawa et al.^192^ found that DNMT3a is upregulated during osteoclastogenesis and contributes to the suppression of anti-osteoclastogenic genes through DNA methylation. This epigenetic silencing facilitates the differentiation of OCPs into mature osteoclasts. Mice with an osteoclast-specific deficiency in DNMT3a exhibit increased bone mass due to a reduced number of osteoclasts, underscoring DNMT3a’s essential role in bone resorption processes^192^. A recent study demonstrated that PU.1 target genes undergo DNA demethylation during the transition from monocytes to osteoclasts^193^. PU.1 cooperates with Tet2, a DNA demethylation enzyme, to remove methyl groups from specific gene promoters, thereby activating genes crucial for osteoclast function^193^. Hypomethylation is accompanied by alterations in 5-hydroxymethylcytosine levels, which represent an intermediate state in the DNA demethylation process^193^. Motif analysis of transcription factor binding sites revealed that the binding motifs for PU.1, NF-κB, and AP-1 (Jun/Fos) were significantly enriched in genes exhibiting DNA methylation changes^193^. Among these, only the PU.1 binding motif showed significant enrichment in both hypermethylated and hypomethylated gene sets^193^. PU.1 was shown to interact with both DNMT3b and TET2, indicating its dual role in facilitating DNA hypermethylation and hydroxymethylation-dependent demethylation^193^. Consistent with these findings, siRNA-mediated PU.1 knockdown in primary monocytes disrupted DNA methylation dynamics and gene expression changes, while also diminishing the recruitment of TET2 and DNMT3b to PU.1 target loci during osteoclast differentiation (Fig. 5)^193^. IRF8 and NFATc1 are also epigenetically regulated during osteoclastogenesis^194^. IRF8 functions as a negative regulator of osteoclast differentiation, and its expression is silenced through DNA methylation, facilitating osteoclastogenesis. In contrast, demethylation of the *Nfatc1* promoter is essential for its activation, promoting osteoclast maturation.

### Histone methylation

Key osteoclast regulatory gene promoters in bone marrow-derived monocytes contain divalent histone modifications that bind activating histone 3 lysine 4-trimethyl (H3K4me3) and inhibitory H3K27me3 markers that break down into inhibitory or activating structures in response to RANKL stimulation^195^. Zeste homolog 2 enhancer (EZH2) is a histone methyltransferase component of multi-comb inhibitory complex 2, catalyzing H3K27me3 modification. RANKL triggers EZH2 translocation into the nucleus and inhibits osteoclast-negative regulators MAFB, IRF8, and ARG1 (Fig. 5)^195^. Inhibition of the RANKL-induced pmTOR-pS6RP pathway by GSK126 alters the translation ratio of C/EBP-β-lap and C/EBP-β-lip isomers and reduces the nuclear transposition of inhibitory C/EBP-β, which is necessary for transcriptional inhibition of osteoclast-negatively regulated transcription factor MAFB^195^. EZH2 in multicellular osteoclasts mainly exists in the cytoplasm^195^. Mature osteoclasts cultured on bone segments with GSK126 showed cytoskeletal defects and decreased absorption activity^195^. EZH2 plays epigenetic and cytoplasmic roles during osteoclast differentiation by inhibiting MAFB transcription and regulating the early stages of PI3K-AKT-mTOR-mediated RANKL signaling^195^.

As the core subunit of H3K4 methyltransferase, Dpy30 is believed to be a key chromatin regulator of cell growth and differentiation and stem cell fate determination. Dpy30 plays an important role in the methylation of H3K4^196–198^. The absence of Dpy30 significantly hindered osteoclast differentiation and inhibited the expression of osteoclast-related genes^199^. In addition, the deletion of Dpy30 reduces the enrichment of H3K4me3 in the promoter region of NFATc1^199^. Deletion of Dpy30 downregulates osteoclast-related genes (such as *CTSK*, *Trap*, *Oscar*, *Car2*, and *Atp6v0d2*). PTIP reduces H3K4me3 on the PPARγ promoter, leading to osteoclast differentiation and impaired function^200^. Furthermore, ARTD1 controls osteoclast occurrence and bone homeostasis by regulating H3K4me3 on the *IL-1β* promoter^201^. Similarly, loss of ASXL1 in bone marrow cells leads to loss of transcriptional inhibitory H3K27me3 and increase of transcriptional activator H3K4me3 in NFATc1, resulting in increased osteoclast accumulation and thus decreased bone mass^202^.

In the presence of ASXL1 defects, increased NFATc1 binds to the *Blimp1* (*Prdm1*) promoter, thereby enhancing the expression of this osteoclast-promoting gene. In AsxL1-deficient osteoclasts, an overall decrease in K27 trimethylation was also accompanied by a 40-fold increase in the expression of the histone demethylase JMJD3^202^. JMJD3 knockdown in AsxL1-deficient OCPs elevates H3K27me3 at the *NFATc1* promoter and inhibits osteoclastogenesis^202^. Thus, in addition to promoting myeloid malignancies, ASXL1 also controls the epigenetic reprogramming of osteoclasts to regulate bone absorption and mass^202^.

The Set1/Mll complex is the major histone H3K4 methylase in mammals. Their complete methylation activity is determined by the relationship between the catalytic subunit and its core subunits Wdr5, Rbbp5, Ash2l, and Dpy30^203^. The expression trend of the core subunit and Set1/Mll complex during osteoclast differentiation is similar to that of Dpy30, suggesting that these genes may also be involved in osteoclast differentiation and need further study^204^. In addition to the main related proteins in the Set1/Mll complex group, Dpy30 also binds to a few other proteins, and thus it cannot be ruled out that Dpy30 regulates osteoclast differentiation through interaction with other factors^204^.

A recent study highlights the pivotal role of PRMT6 in the epigenetic regulation of metabolism during osteoclast differentiation^205^. Activation of RANK signaling induces PRMT6 upregulation through epigenetic modification, initiating a metabolic switch from fatty acid oxidation to glycolysis. This metabolic reprogramming is essential for osteoclastogenesis, as PRMT6-mediated glycolysis supports the energy-intensive process of osteoclast differentiation^205^. In contrast, loss of Prmt6 reverses this metabolic shift by enhancing fatty acid oxidation and suppressing hypoxia-inducible factor 1-alpha (HIF1α)-mediated glycolysis, thereby impairing osteoclast formation and alleviating bone loss in OVX mice^205^. PRMT6 deficiency reduces asymmetric dimethylation of H3R2 on the promoters of key metabolic genes such as PPARδ, Acox3, and Cpt1a, thereby enhancing chromatin accessibility and promoting fatty acid oxidation^205^. This positions PRMT6 as a metabolic checkpoint that drives osteoclast differentiation through its epigenetic influence on metabolic pathways. Targeting PRMT6 offers a novel therapeutic strategy to modulate osteoclastogenesis and combat pathological bone resorption^205^.

### Histone acetylation

Histone deacetylase 1 (HDAC1) acts as an early transcriptional repressor in osteoclast differentiation, showing high expression in precursors but being markedly downregulated after RANKL stimulation^206,207^. HDAC1 mainly functions as a transcriptional corepressor by binding to the promoters of osteoclast-related genes and inhibiting their expression^206,207^. ChIP analyses further demonstrate that upon M-CSF stimulation, MITF and PU.1 are recruited to the *Ctsk* and *Acp5* promoters together with co-repressors including CTBP, Sin3A, and HDAC1^206,207^. In contrast, HDAC2 expression increases during osteoclastogenesis^208^. Functional studies reveal that HDAC2 knockdown not only reduces osteoclast differentiation but also impairs cytoskeletal organization, cell fusion, and resorptive activity^208^. HDAC2 has been shown to activate Akt, which in turn suppresses the inhibitory effect of FoxO1, indicating that HDAC2 functions as a positive regulator rather than an inhibitor of osteoclast differentiation (Fig. 5)^208^.

HDAC3 is expressed in OCPs but is maintained at low levels during differentiation. Knockdown of HDAC3 inhibits osteoclastogenesis by reducing the expression of NFATc1, CTSK, and Dc-stamp, while simultaneously promoting osteoblast differentiation^209,210^. Loss of HDAC3 function mimics the effects of broad-spectrum HDAC inhibitors, including trichostatin A (TSA) and sodium butyrate (NaB)^209,210^.

HDAC5 is expressed at later stages of osteoclast differentiation, with its expression peaking during the cell fusion phase^211^. Similar to HDAC4, shRNA knockdown of HDAC5 resulted in increased osteoclast differentiation, upregulation of osteoblast genes, and increased absorption^211^. Mouse global deletion of HDAC5 was not severely defective at birth and the mouse size was normal^212^. Global deletion of HDAC5 affects multiple bone cell types, and the underlying mechanism of bone loss remains unclear, highlighting the need for tissue-specific studies^212^. These mechanisms are being investigated using osteoclast-specific HDAC5 knockout models^212^. Mechanistically, HDAC5 deacetylates NFATc1, promoting its instability, which may represent a key mechanism through which HDAC5 suppresses osteoclast differentiation^212^.

During osteoclast differentiation, the class IIb deacetylase HDAC6 reaches peak expression at the fusion stage and is primarily localized in the cytoplasm^213,214^. shRNA-mediated knockdown of HDAC6 in BMDMs produced no detectable phenotype and did not affect the expression of major osteoclast genes. Instead, HDAC6 primarily regulates cytoskeletal organization by destabilizing the osteoclast actin network, thereby limiting cell migration and podosome belt formation^213,214^. Mechanistically, HDAC6 associates with RhoA and mDia2 to regulate podosome organization. Microinjection of activated RhoA or mDia2 promotes microtubule deacetylation and disrupts the structural integrity of the podosome belt^213,214^.

Sirtuins critically regulate osteoclast differentiation and activity via distinct mechanisms. Sirtuin 1 (SIRT1) suppresses osteoclastogenesis by inhibiting RANKL signaling, primarily through deacetylation and activation of Forkhead box (FOX) proteins, which function as osteoblast inhibitors^215^. Loss of SIRT1 increases acetylation of FOX proteins, thereby enhancing osteoclast differentiation and resorptive activity^215^. SIRT6 is expressed in osteoclasts in response to M-CSF and RANKL stimulation and inhibits NF-κB transcription via deacetylation of H3K9, leading to suppression of NF-κB target genes^216^. Overexpression of SIRT6 inhibits osteoblast generation, while its loss enhances osteoblast differentiation^216^. Together, SIRT1, SIRT3, and SIRT6 exhibit nuanced regulatory roles in osteoclast biology, influencing differentiation and activity through distinct molecular pathways.

### Small RNA

Small RNAs, including microRNAs (miRNAs) and other non-coding RNAs, play crucial regulatory roles in osteoclast differentiation by modulating gene expression at the post-transcriptional level. During osteoclastogenesis, specific miRNAs act as key regulators, either promoting or inhibiting the differentiation process. For example, miR-124 and miR-148a inhibit osteoclast differentiation by targeting RANK signaling and other osteoclastogenic pathways, whereas miR-21 and miR-223 facilitate osteoclast formation by upregulating key transcriptional regulators such as c-Fos and NFATc1 (Fig. 5). Additionally, long non-coding RNAs (lncRNAs) and circular RNAs (circRNAs) often act as competing endogenous RNAs (ceRNAs) to sequester miRNAs, thereby indirectly regulating osteoclastogenesis. The delicate balance of small RNA activity is critical for maintaining bone homeostasis, and dysregulation of these molecules can lead to pathological conditions such as osteoporosis or RA. Recent studies have highlighted the therapeutic potential of targeting small RNAs to modulate osteoclast activity and treat bone resorption diseases.

A recent study investigates the role of osteoclast-derived exosomes and the molecular mechanism of the lncRNA AW011738/miR-24-2-5p/TREM1 axis in osteoporosis^217^. Exosomes derived from RANKL-induced osteoclasts have been shown to be enriched in lncRNA AW011738, which plays a crucial role in suppressing osteoblast differentiation. AW011738 suppresses osteogenesis-related markers by regulating TREM1 expression via miRNA interactions, particularly miR-24-2-5p, which downregulates TREM1 to promote osteoblast differentiation^217^. Knockdown of AW011738 in osteoclast-derived exosomes increased osteoblast differentiation markers and reduced bone loss in OVX mice, as evidenced by micro-CT analysis. In vitro osteoclast-derived exosomes with silenced AW011738 promoted osteoblast differentiation, whereas exosomes with overexpressed AW011738 inhibited this process^217^. Bioinformatics and protein–protein interaction analyses supported the regulatory role of this axis in bone metabolism. Furthermore, immune microenvironment changes linked to AW011738 upregulation were identified. These findings highlight that osteoclast-derived exosomes carrying lncRNA AW011738 exacerbate osteoporosis by inhibiting osteogenesis through the AW011738/miR-24-2-5p/TREM1 pathway, offering a potential therapeutic target for mitigating bone loss in osteoporosis^217^.

The bone microenvironment contributes additional inhibitory cues that fine-tune osteoclast activity. Semaphorin 3A (Sema3A) suppresses osteoclast formation through Neuropilin-1-mediated inhibition of RhoA and NFATc1 signaling^218^. At the post-transcriptional level, inhibitory miRNAs (e.g., miR-124^219^, miR-146a^220^, miR-155^221^, miR-223^222^) target key osteoclastogenic genes such as TRAF6, NFATc1, and MITF, attenuating differentiation and resorptive function. These extracellular and epigenetic brakes maintain the balance between bone resorption and formation.

Beyond small RNA-mediated regulation, N6-methyladenosine (m⁶A) RNA methylation is also a crucial post-transcriptional modification that influences osteoclast differentiation. A recent study reported that FTO, a crucial demethylase of m⁶A, regulates OCP proliferation and apoptosis through m⁶A RNA demethylation, thereby promoting osteoclastogenesis^223^. This highlights the role of RNA modifications beyond small RNA interactions in controlling osteoclast activity and bone homeostasis.

## Cytokine and growth factors regulating osteoclast differentiation and activation

Cytokines and growth factors such as RANKL, M-CSF, TGF-β, and parathyroid hormone (PTH) serve as key regulators of osteoclast differentiation and activation. These factors influence bone remodeling by modulating osteoclastogenesis, often in concert with osteoblast activity, leading to bone resorption or formation depending on the balance of these signals.

### M-CSF

M-CSF plays an important role in supporting the proliferation and survival of osteoclast progenitors. The binding of M-CSF to its homologous receptor c-Fms results in autophosphorylation and reverse phosphorylation of specific tyrosine residues in the cytoplasmic tail of c-Fms^224,225^. Four of the eight tyrosine residues in the cytoplasmic tail of c-Fms (Y544, Y559, Y697, Y706, Y721, Y807, Y921, Y974) have been shown to functionally regulate the proliferation and survival of osteoclast progenitors^224,225^. Phosphorylation of Y559 is essential for the full activation of c-Fms. Once phosphorylated, Y559 interacts with c-Src to form a signaling complex that recruits PI3K and c-Cbl^224,225^. This assembly activates the Akt pathway and simultaneously promotes c-Fms ubiquitination, thereby coupling receptor activation with downstream signaling and receptor turnover^224,225^. c-Cbl-dependent c-Fms ubiquitination enhances tyrosine phosphorylation and activation through conformational changes in the kinase domain. Phosphorylated Y721 also activates the Akt pathway through direct interaction with PI3K^224,225^. In contrast, phosphorylated Y697 and Y974 interact with Grb2 to activate ERK signaling^226^. Thus, M-CSF-induced activation of c-Fms promotes OCP proliferation and survival via the ERK and PI3K/Akt pathways. Phosphorylation of Y544 and Y807 is also essential for c-Fms activation and osteoclast differentiation, although their binding partners and precise downstream mechanisms remain undefined. Analysis of mice lacking *csf1r* (the gene encoding c-Fms) also supported the critical role of M-CSF in osteoclast differentiation in mice that exhibited bone atypia (Table 1).

Upon binding to its receptor C-FMS, M-CSF recruits adaptor proteins and cytosolic kinases, thereby initiating a series of intracellular signaling cascades^227^. Within the cytoplasmic domain, tyrosine residues Y559 and Y807 exert distinct roles in osteoclast differentiation and function^227^. C-FMS expression governs the lineage commitment of monoblastic progenitors, while M-CSF-induced transcriptional programs are crucial for maintaining cellular responsiveness to RANKL and interleukins^227^. M-CSF upregulates RANK and multiple RANK/NF-κB pathway components, including TRAF2A, PI3K, MEKK3, and RIPK1, thereby providing a mechanistic basis for M-CSF-RANKL synergy^228^. Beyond its differentiation role, M-CSF also serves as a survival factor for OCPs by upregulating Bcl-xL, which inhibits caspase-9 activation and prevents apoptosis^228^.

M-CSF promotes OCP survival primarily through Grb2- and Akt-dependent signaling, activating ERK via the PI3K pathway^229^. M-CSF also induces RANK expression in BMDMs, thereby enabling effective responsiveness to RANKL^229^. Transcription factors essential for osteoclast progenitor proliferation and survival are closely coupled to M-CSF signaling^229^. Notably, the anti-apoptotic factor Bcl-2 is a direct transcriptional target of MITF, and Bcl-2-deficient mice display an osteopetrotic phenotype (Table 1)^229^. Overexpression of Bcl-2 rescues this phenotype in op/op mice, underscoring the pivotal role of the M-CSF/c-Fms axis in sustaining osteoclast progenitor survival through Bcl-2-mediated anti-apoptotic signaling^229^.

### RANKL

RANKL, also known as TNF-related activation-induced cytokine (TRANCE), osteoprotegerin ligand (OPGL), or osteoclast differentiation factor (ODF), is a member of the TNF superfamily^230^. Genetic ablation of RANK results in severe osteopetrosis due to a complete block in osteoclast differentiation, underscoring the indispensable role of RANK signaling in osteoclastogenesis (Table 1)^231^. Upon binding of RANKL to its receptor RANK, TRAF6 is recruited, which in turn activates the downstream NF-κB signaling cascade. This activation promotes the nuclear translocation of NF-κB and subsequently initiates the transcription of genes required for osteoclast differentiation^232^. In addition, MAPKs, including ERK, JNK, and p38, become activated and subsequently stimulate downstream transcription factors such as c-Fos and AP-1, both of which are essential for the process of osteoclast differentiation (Fig. 2)^233^. OPG, encoded by the Tnfrsf11b, functions as a soluble decoy receptor for RANKL and is expressed in both osteoclasts and pre-osteoclasts. By binding RANKL, OPG blocks its interaction with RANK, thereby inhibiting osteoclastogenesis. Dysregulation of OPG disrupts bone homeostasis: overexpression causes osteoclast-deficient osteopetrosis, whereas deficiency leads to osteoporosis due to excessive osteoclast formation and activity (Table 2). RANKL is expressed in activated T cells and may have the ability to act directly on osteoclast progenitors under pathological conditions^106^. On the other hand, IFN-γ produced by T cells even at tiny concentrations inhibits RANKL-induced osteoclast differentiation by activating the ubiquitin-proteasome system to accelerate the degradation of TRAF6^106^.

The RANKL:OPG ratio serves as a key regulatory determinant of osteoclastogenesis. Various cell types, including immune cells, modulate this balance to control osteoclast differentiation and bone resorptive activity^234,235^. RANKL serves as the principal ligand driving osteoclast formation, whereas OPG acts as its competitive inhibitor (Fig. 7). Immune cells contribute to osteoclast regulation not only by directly producing RANKL, thereby elevating the RANKL:OPG ratio, but also by modulating RANKL expression in osteoblasts and secreting inflammatory cytokines that act either directly or indirectly on osteoclasts. Thus, pro-inflammatory cytokines secreted by immune cells, including macrophages and dendritic cells (DCs), such as TNF-α, IL-6^236^, interleukin-23 (IL-23), and IL-1β, serve as key drivers of osteoclast activation and differentiation (Fig. 7)^237^. Immune cells can also inhibit osteoclasts by releasing cytokines such as interleukin 4 (IL-4), interleukin-18 (IL-18), interleukin-33 (IL-33), and IFN^235^. The influence of these inflammatory cytokines is critical for the regulation of osteoclasts after fracture^99,238^.

The IVVY motif of RANK regulates the expression of key osteoclast genes, including NFATc1, C/EBPα, CTSK, PU.1, and TRAP, and promotes osteoclast differentiation driven by TNF-α and IL-1 (Table 2 and Fig. 4)^239,240^. In addition, IVVY motifs are also essential for osteoclastogenesis^241,242^. Previous research has demonstrated that the inactivation of IVVY motifs of RANK inhibits C/EBPα activity, but IVVY motifs have no significant effect on c-Fos activity^185^. However, the RANK IVVY motif alone may also be insufficient to upregulate C/EBPα during osteogenesis, as the IVVY motif has been reported to induce osteoclast genes during osteoclast differentiation by cooperating with two other RANK cytoplasmic motifs (_559_PVQEET_564_ and _604_PVQEQG_609_)^243^. Thus, IVVY motifs may act in conjunction with other RANK motifs to upregulate C/EBPα during osteoclast differentiation (Fig. 4)^244^. The inactivation of the IVVY sequence of RANK can lead to the increase of mRNA level of osteoclast differentiation inhibitor RBP-J, and further inhibit the down expression of osteoclast differentiation-related genes, such as NFATc1 and C/EBPα^11^. RBP-J inhibits ITAM-mediated cost-effective signaling and limits crosstalk between ITAM and RANK signals, which ultimately inhibits osteoclast differentiation by inhibiting Ca^2+^-CaM signaling pathways^245^.

RANKL can regulate the differentiation process of osteoclasts by regulating downstream Guanine nucleotide-binding protein subunit α_13_ (Gα_13_). Gα_13_-RhoA signaling negatively regulates osteoclastogenesis by suppressing the Akt-GSK3β-NFATc1 axis^246^. Downstream of RANK and c-Fms, Akt phosphorylates and inactivates GSK3β, thereby facilitating NFATc1 nuclear retention and promoting osteoclast differentiation. In contrast, Gα_13_ activates RhoA, which attenuates Akt phosphorylation and activity, counteracting excessive osteoclast activation^246^. Notably, Gα_13_ expression is induced by combined RANKL and M-CSF stimulation, serving as a feedback mechanism to limit osteoclast differentiation, protect viable bone cells, and prevent excessive bone loss^246^. As such, the Gα_13_-RhoA-Akt-GSK3β-NFATc1 signaling cascade can negatively affect osteoclast formation and osteoclast function^246^.

### RANKL and M-CSF as key osteoclastogenic factors

M-CSF and RANKL are essential for osteoclast differentiation, with research highlighting their role in regulating osteoclastogenesis and bone remodeling (Fig. 7). The RANKL-RANK-TRAF6 signaling cascade is the fundamental driver of osteoclast differentiation. RANKL, produced by osteoblasts, osteocytes, and stromal cells, binds to its receptor RANK on monocyte/macrophage lineage precursors, triggering recruitment of the adaptor molecule TRAF6^232^. Disruption of RANK, RANKL, or TRAF6 in mice results in severe osteopetrosis, confirming the indispensability of this pathway in skeletal homeostasis^247^.

Clark et al.^248^ investigated intrinsic differences in osteoclast progenitors from the mouse mandible and femur, revealing that M-CSF-driven differentiation potential varies by region, influenced by localized factors and cytokines. Similarly, Cheng et al.^249^ emphasized the importance of M-CSF and RANKL in osteoclast formation and demonstrated how modifying interactions between osteoclasts and other bone cells, such as osteoblasts, can impact bone resorption and regeneration, with targeted delivery of growth factors offering therapeutic potential. Lacey et al.^250^ produced seminal work confirming that RANKL is indispensable for osteoclast differentiation through its interaction with RANK on OCPs, forming the basis for many osteoclast-targeting therapies (Fig. 7). Zhao’s study further supported the centrality of M-CSF in osteoclastogenesis by identifying bone marrow Adiponectin (Adipoq) lineage progenitors as the primary source of M-CSF (Fig. 7)^251^. Expanding on this, Zhong et al.^252^ demonstrated that bone marrow adipogenic precursors are not only critical for M-CSF production but also for hematopoiesis, underscoring the significance of bone marrow microenvironments in supporting osteoclast activity and overall bone health.

### Interleukins

Interleukin is a lymphoid factor that interacts with white blood cells or immune cells. They coordinate and interact with each other to complete hematopoietic and immunomodulatory functions. Interleukin plays an important role in message transmission, activation and regulation of immune cells, osteoclast differentiation, and inflammation^253,254^.

Interleukin-10 (IL-10) can inhibit osteoclast differentiation^255^. Studies have shown that IL-10 can methylate MEG3, thereby inhibiting the expression of MEG3^138^. MEG3 promotes osteoclast differentiation by competing with IRF8 to bind STAT1, preventing the interaction between IRF8 and STAT1, thereby dampening the inhibitory effect of IRF8 on osteoclast differentiation^138^. In vitro studies have shown that exogenous IL-10 can downregulate the expression of MEG3 and upregulate the expression of IRF8 in RANKL-mediated osteoclast differentiation, thus inhibiting osteoclast differentiation^256^. In vitro studies have shown that IL-33 can inhibit osteoclast differentiation driven by NF-κB by directly inhibiting M-CSF receptor activators^257^. Interleukin-3 (IL-3) inhibits RANKL-mediated osteoclast differentiation by downregulating the activation of the NF-κB signaling pathway^258^ or inhibiting TNF-α-mediated osteoclast differentiation by downregulating the expression of TNF receptors 1 and 2^259^. In addition, IL-3 inhibits the synergistic effect of TNF-α with RANKL, IL-1β, TGF-β1 and TGF-β3^260^. It suggests that IL-3 also plays an important role in bone loss-related diseases^260^.

IL-1 is an important cytokine that promotes osteoclast differentiation^253^. However, studies have shown that although IL-1 can activate early signaling pathways such as NF-κB, JNK, and p38, IL-1 alone is not sufficient to induce differentiation of OCPs^57^. The expression of IL-1RI and c-Fos induced by RANKL or TNF-α may lead to the formation of IL-1-induced osteoclasts^253,261^. IL-1 may drive osteoclast differentiation through RANKL/RANK-independent mechanism^262^. IL-1 can directly induce osteoclast through IL-1RI receptor, and osteoclast-related transcription factors RNAKL and TNF-α can also upregulate the expression of IL-1RI receptor through c-Fos and NFATc1^262^. In addition, IL-1 partially activates osteoclast-specific genes, including TRAP and OSCAR, through the MITF pathway^262^. Regulatory T cells (Tregs) can express IL-1 receptor IL-1R, and thus IL-1β can produce O-Tregs and cause bone erosion in inflammatory arthritis^263^. Tregs can also limit joint destruction by directly inhibiting osteoclast development through contact-dependent and -independent mechanisms^264–266^. As such, IL-1β can accelerate osteoclast differentiation by driving RNAKL expression in Tregs^267^.

In addition, IL-6 also promotes osteoclast differentiation. It has been shown that IL-6 can stimulate RNAKL expression in osteoblasts through JAK2/STAT3 pathway^268^, and IL-6 can also induce RANKL expression by activating STAT3 in osteoblasts^269^, thus promoting osteoclast differentiation. When IL-6 binds to its receptor (IL-6R), it triggers homodimerization of the signal transduction coreceptor glycoprotein 130 (gp130), leading to the activation of downstream signaling pathways, such as Janus kinase/signal transducer and activator of transcription (Jak/STAT), PI3K, and MAPK cascades^270^. Recent studies have shown that zoledronate enhances osteoblast-mediated osteoclast formation by enhancing IL-6 expression and subsequent RANKL expression^271^. Recombinant gp130 can also selectively block the promoting effect of IL-6 on the downstream RANKL signaling pathway without affecting IL-6 classical signaling^272^. Therefore, the inhibitory effect of recombinant gp130 on the promotion of IL-6 on osteoclast differentiation at low RANKL level may provide an effective therapeutic approach for the treatment of inflammatory and metabolic osteolytic diseases such as RA and postmenopausal osteoporosis.

## Osteoclast function and bone resorption

### Bone resorption

**Fig. 6:**
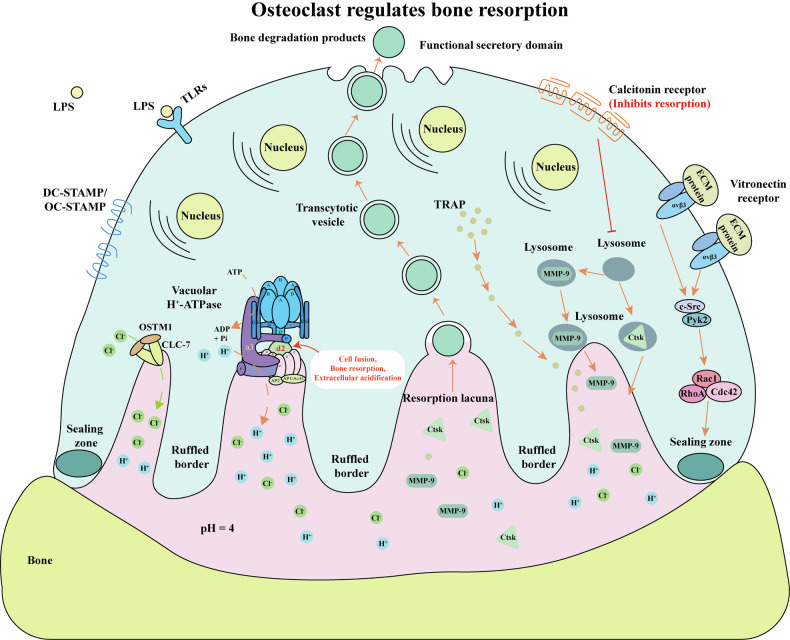
Osteoclast-mediated bone resorption. At early stages, OCPs express DC-STAMP and OC-STAMP, two essential transmembrane proteins required for precursor cell fusion and multinucleated osteoclast formation. Once mature, osteoclasts adhere tightly to the calcified bone surface via the vitronectin receptor (αvβ3 integrin), which recognizes matrix proteins containing RGD motifs, ensuring firm attachment and forming a sealing zone — an actin ring that isolates the bone resorption zone. The ruffled border is a highly convoluted membrane structure on the bone-facing side of the osteoclast that is rich in vesicles and proton pumps. The vacuolar H^+^-ATPase actively transports protons into the resorption cavity, creating an acidic microenvironment (pH ~4.5), which is required for the dissolution of the inorganic mineral matrix (hydroxyapatite). This acidification process is coupled to ClC-7, a chloride channel that maintains ionic balance during proton transport. In this acidic environment, osteoclasts secrete matrix-degrading enzymes, including TRAP, CTSK and MMP-9. The CTR on the surface of osteoclasts can act as a negative regulator of resorption, responding to calcitonin signaling by inhibiting osteoclast activity and disrupting cytoskeletal organization. Meanwhile, the cytoskeleton, especially the actin ring in the sealing zone, is tightly regulated by the integrin αvβ3-c-Src-Pyk2 signaling axis and cooperates with Rac, Rho, and Cdc42 to ensure proper adhesion and polarization. Osteoclasts also exhibit bactericidal properties through phagocytosis, ROS production, and acidification. TLRs can recognize LPS to modulate osteoclast activity in both bone resorption and antimicrobial defense.

Bone resorption, the primary function of osteoclasts, represents one component of the coupled bone remodeling cycle that maintains skeletal homeostasis. Osteoclast activation initiates with adhesion to the bone matrix, followed by cytoskeletal reorganization, cell polarization, and the development of specialized resorptive membrane domains. During this process, actin filaments assemble into a characteristic ring-shaped sealing zone, which anchors the osteoclast tightly to the mineralized bone surface and creates a confined microenvironment for matrix degradation (Fig. 6). CTSK is the main protein responsible for bone resorption^27,263,264^. CTSK plays a critical role in bone resorption by degrading type I collagen, a primary component of the bone matrix in low pH condition^265,273^. Osteoclast activity is tightly regulated by cytokines. For example, IL-1α enhances osteoclast-mediated bone resorption by upregulating CTSK expression through activation of the NF-κB signaling pathway^266,267^. Conversely, IFN-γ exerts an inhibitory effect on osteoclast differentiation at multiple stages, not only suppressing early osteoclast differentiation but also downregulating CTSK production, thereby reducing the late-stage bone-resorbing activity of osteoclasts^266^.

MMP-9 is highly expressed in osteoclasts and plays a crucial role in bone matrix degradation^27^. During bone resorption, activated osteoclasts secrete MMP-9 into the resorption lacuna, where it contributes to the breakdown of type I collagen and other extracellular matrix proteins that compose the organic phase of bone^274^ (Fig. 6). In addition to its proteolytic activity, MMP-9 facilitates osteoclast migration and invasion by remodeling the extracellular environment, thereby promoting the expansion of resorption sites. Moreover, MMP-9 activity is tightly regulated by cytokines and signaling pathways such as RANKL-NF-κB and MAPK, underscoring its essential function in osteoclast-mediated bone remodeling and in pathological bone loss conditions such as osteoporosis, arthritis, and tumor-induced osteolysis.

Additionally, osteoclasts play a dual role in bone remodeling by promoting osteoblast activity and by releasing factors such as TGF-β, insulin-like growth factor 1 (IGF-1), and Wnt signaling inhibitors during resorption^34,268–273^. Osteoclasts are also involved in influencing systemic metabolism through secreted factors, such as dipeptidylpeptidase-4 (DPP4), which reduces GLP-1 levels, leading to decreased insulin, increased glucagon secretion, and hyperglycemia, linking bone remodeling to energy homeostasis^34,268–272^. As can be seen, osteoclasts are involved in an array of physiological functions, including bone resorption, regulation of bone formation, and systemic energy metabolism.

Our laboratory provided evidence that the vacuolar-type proton-translocating ATPase (V-ATPase) subunit Atp6i (OC-116 kDa, a3, also known as Atp6v0a3) is a unique and essential component of osteoclast-mediated extracellular acidification, functioning as a vacuolar proton pump (Fig. 6 and Tables 1 and 2)^275–277^. We demonstrated that the V-ATPase subunit Atp6v1c1 (C1) was highly expressed in osteoclasts^278^, while the Atp6v1c2a (C2a) and Atp6v1c2b (C2b) subunits were not^279^. We demonstrated that the V-ATPase subunit ATP6AP1 (Ac45) regulates multiple aspects of osteoclast function, including differentiation, extracellular acidification, lysosomal trafficking, and protease exocytosis during bone resorption^280,281^. During osteoclast differentiation, the expression level of C1 is highly induced by the activator for NF-κB ligand RANKL and M-CSF^280,281^. C1 interacts with a3 and is mainly localized in the fold margin of activated osteoclasts. C1 is an important component of the frillated proton pump of osteoclasts and may regulate the formation of F-actin rings during osteoclast activation^279^. In addition, the expression of the d subunit Atp6v0d2 (d2) isoform of V-ATPase was 5 times higher in mature osteoclasts than d1 isoform, indicating a potential function in osteoclast bone resorption. We demonstrated that the d2 subunit is essential for V-ATPase-mediated proton pumping at the osteoclast ruffled border, likely through its direct interaction with the N-terminus of the a3 subunit. This unique d2–a3 complex plays a critical role in osteoclast function, and our results suggest that d2 may represent a promising therapeutic target for osteoporosis due to its key involvement in regulating osteoclast-driven extracellular acidification during bone resorption (Fig. 6)^282^.

Bone resorption begins when the integrin αVβ3 enables the attachment of osteoclasts to the bone surface, thereby recruiting Src tyrosine kinase within osteoclasts through external signaling (Fig. 6)^224,283^. This process activates multiple Src-dependent pathways, in which Src phosphorylates Syk, promoting the recruitment of DAP12, ITAM co-stimulatory proteins, and Slp76 to form an adaptor complex that subsequently activates Rho family GTPases such as Rac^225,283^. αVβ3 physically interacts with M-CSFR, promoting integrin activation through both inside-out and outside-in signaling. Similarly, RANK interacts with αVβ3 through Src to activate downstream signaling molecules such as Syk, Slp-76, Vav3, and Rac, mirroring the αVβ3/Src signaling cascade^226^. These interactions lead to the fusion of “secretory lysosomes” with the cytoplasmic membrane to form the osteoclast-specific ruffled border for secreting protons (H^+^), chloride ions (Cl^−^) and lysosomal hydrolases (e.g., CTSK) to digest the exposed organic matrix^284^. Vesicle trafficking and fusion in osteoclasts is coordinated by multiple proteins, including Rab7, Rac1, synaptotagmin VII, PLEKHM1, LC3, GSDMD, and SNX10, integrating endocytic, secretory, transcytotic, and autophagic pathways (Table 2)^12,285–288^.

The intricate molecular mechanisms driving osteoclast-mediated bone resorption not only highlight its importance in bone remodeling but also present valuable therapeutic targets for bone diseases and cancer metastasis. During bone resorption, osteoclasts acidify the resorption lacuna by actively transporting H^+^ ions across the ruffled border. These protons combine with Cl^−^ ions to form hydrochloric acid (HCl), which dissolves and demineralizes the inorganic components of the bone matrix (Fig. 6). This acidification is essential because it creates an optimal acidic environment that allows CTSK, a key protease, to efficiently degrade the organic components of the bone matrix. CTSK inhibitors are currently being explored as potential antiresorptive therapies, and recent findings indicate that osteoclast-expressed CTSK can suppress RANKL expression in osteoblasts, highlighting its role in bone remodeling. Additionally, the Src kinase, which is essential for osteoclast resorption activity, also plays a key role in cancer cell proliferation, invasion, and metastasis. Src inhibitors, such as saracatamib, are under investigation as adjunct therapies in treating metastatic prostate cancer. These molecular mechanisms and their potential inhibitors offer promising therapeutic avenues for both bone diseases and cancer metastasis^35,277,289^.

During bone formation, proteins such as TGF-β, BMPs, fibroblast growth factors (FGFs), and IGFs are incorporated into the collagen matrix and subsequently released by osteoclasts during bone resorption. Once liberated, these factors can be activated and locally utilized to modulate bone remodeling under both physiological and pathological conditions, including metastatic bone disease^290,291^. For example, TGF-β, BMP, FGF, and IGF may regulate osteoblast differentiation by directly and/or indirectly regulating the expression of RANKL or OPG. TGF-β also acts as a coupling factor to attract osteoblasts to the absorption site^35^.

A recent study indicated that caspase-8 activation plays a critical role in osteoclast fusion and bone resorption^292^. The formation of multinucleated osteoclasts, essential for bone resorption, is initiated by RANKL-induced activation of caspase-8 in mononuclear OCPs^292^. Single-cell RNA sequencing revealed that osteoclast differentiation involves partial activation of apoptotic pathways^292^. Caspase-8 activation cleaves downstream effector caspases, such as caspase-3, which then activate the phospholipid scramblase Xkr8^292^. Xkr8 facilitates the exposure of phosphatidylserine on the plasma membrane, enabling recognition by fusion partners and promoting the formation of functional osteoclast syncytia. Pharmacological or genetic inhibition of caspase-8 disrupts osteoclast fusion and bone resorption, resulting in increased bone mass in mice^292^. By identifying that caspase-8 and phosphatidylserine exposure are key regulators of osteoclast biology, potential therapeutic targets for conditions involving pathological bone loss can be investigated with this mechanism in mind.

### Discovery and characterization of osteoclast function genes

Osteoclast function genes have been discovered and characterized through a combination of advanced molecular, genetic, and cellular techniques. Researchers initially identified these genes by comparing gene expression profiles of osteoclasts with other cell types using transcriptomics, revealing osteoclast-specific genes such as CTSK^293^, ACP5, RANK, and its ligand RANKL^294^. Proteomic studies further validated these findings by detecting proteins uniquely expressed in osteoclasts, such as lysosomal enzymes critical for bone matrix degradation. Functional validation using gene knockout and transgenic mouse models demonstrated the necessity of these genes for osteoclast differentiation and activity. For instance, mice lacking CTSK exhibit impaired bone resorption, while RANK and RANKL knockout models develop severe osteopetrosis due to defective osteoclastogenesis^295^. In vitro assays using bone marrow-derived precursors combined with RNA interference or CRISPR-Cas9 editing have been instrumental in delineating the roles of these genes in differentiation and bone resorptive activity^296^. Additionally, pathway analyses revealed critical signaling cascades such as the RANKL-RANK-OPG axis and lysosomal pathways, further linking these genes to osteoclast function^297^.

**Fig. 7:**
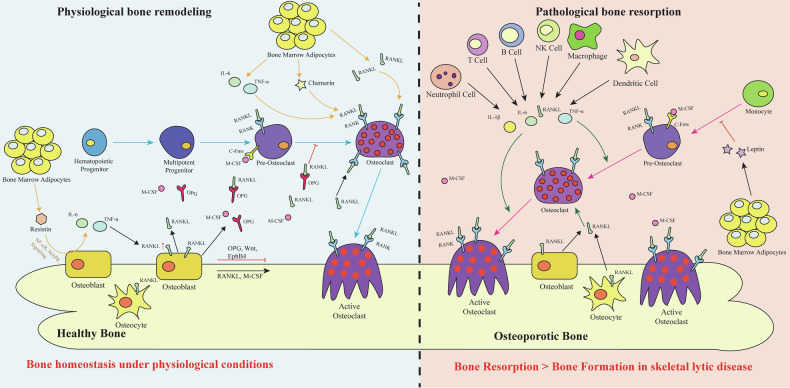
Regulation of osteoclast differentiation by bone marrow adipocytes, osteoblasts, and immune cells under physiological and pathological bone remodeling conditions. During physiological bone remodeling (left), bone marrow adipocytes secrete factors such as RANKL, IL-6, TNF-α, Resistin, and Chemerin, all of which promote osteoclast differentiation by activating NF-κB and MAPK signaling pathways. In addition, osteoblasts and osteocytes promote osteoclastogenesis by expressing RANKL, while osteoblast-derived M-CSF supports the survival and proliferation of OCPs. To balance excessive osteoclast formation, osteoblasts also secrete OPG, which acts as a decoy receptor for RANKL, thereby inhibiting osteoclast differentiation. In addition, Wnt signaling and EphrinB2-EphB4 signaling from osteoblasts also inhibit osteoclast differentiation and help maintain bone homeostasis. Transcription factors Runx2 and Cbfβ regulate osteoblast differentiation and function, ensuring normal bone formation. This balancing act maintains bone health. In pathological bone resorption (right), various immune cells (including T cells, B cells, NK cells, macrophages, DCs, and neutrophils) are activated and release inflammatory cytokines such as IL-6, TNF-α, IL-1β and RANKL. These factors overstimulate osteoclast differentiation and activation, tipping the balance toward increased bone resorption. The resulting imbalance (bone resorption exceeding bone formation) leads to osteoporosis, which is characterized by increased porosity and fragility. Bone marrow adipocytes also participate in the regulation of pathological bone resorption. Bone marrow adipocytes can secrete leptins, which can inhibit the process by which monocytes differentiate into pre-osteoclasts.

Specific genes have been associated with various diseases. In osteoporosis, RANKL, OPG, and sclerostin are key regulators, with sclerostin inhibiting bone formation (Table 2)^298^. In periodontal diseases, MMP-9 and IL-6 play roles in inflammatory-driven bone loss, while TNF-α and TRAP (ACP5) are implicated in osteoclast activation^299^. Endodontic diseases often involve MMP-8 and RANK, which mediate periapical bone resorption^300^. RA is strongly linked to TNF-α, IL-1β, and RANKL, all of which drive osteoclastogenesis and joint destruction (Fig. 7)^301^. Inflammatory bone diseases frequently involve IL-17, CXCL8, and NFATc1, highlighting the interaction between inflammatory cytokines and osteoclast-related genes^302^.

Mouse models have been instrumental in understanding these diseases and their underlying mechanisms. For osteoporosis, RANK and RANKL knockout mice have provided critical insights, with severe osteopetrosis observed due to impaired osteoclast differentiation (Table 1)^295^. In periodontal diseases, models overexpressing IL-6 or TNF-α mimic inflammatory bone loss, while MMP-9 knockout mice display reduced periodontal degradation^299^. Endodontic disease models using RANK overexpression show enhanced periapical bone resorption, providing targets for potential therapies^300^. RA studies rely heavily on TNF-α transgenic mice, which replicate joint destruction and inflammation, while inflammatory bone disease models featuring IL-17 and NFATc1 knockouts reveal their essential roles in osteoclast regulation and cytokine interplay^301^.

## Osteoclastogenisis in inflammatory conditions

Inflammatory conditions have been shown to significantly influence the process of osteoclastogenesis, leading to enhanced bone resorption and associated pathologies^303^ (Fig. 7). Recent studies have shed new light on the mechanisms through which inflammation promotes osteoclastogenesis, emphasizing the contributions of diverse cytokines, signaling pathways, and intercellular interactions^304,305^. Inflammatory osteoclasts (iOCs) are a distinct subset of bone-resorbing cells that arise under chronic inflammatory conditions, including RA and periodontitis. Unlike classical osteoclasts, iOCs originate from diverse precursors, including monocytes, DCs, and arthritis-associated osteoclastogenic macrophages, and exhibit a distinct proinflammatory phenotype^306,307^. These cells not only resorb bone but also produce cytokines such as IL-1β, IL-6, and TNF-α, further amplifying local and systemic inflammation^8^ (Fig. 7). In RA, iOCs contribute to synovial inflammation and systemic bone erosion, while in periodontitis, microbial dysbiosis triggers their activation, leading to alveolar bone destruction^307,308^. Interestingly, iOCs can act as antigen-presenting cells, linking innate and adaptive immune responses, and perpetuating tissue damage. Shared pathogenic mechanisms between RA and periodontitis underscore their potential as therapeutic targets. Targeting iOCs with therapies such as cytokine inhibition (e.g., TNF-α, IL-6) or modulating their precursors could mitigate pathological bone resorption while preserving physiological bone remodeling^306,307^.

### Inflammatory cytokines in inflammatory osteoclastogenesis

Inflammatory cytokines such as TNF-α, IL-1^7^, and IL-6^8^ play a pivotal role in osteoclastogenesis. These cytokines can stimulate the expression of RANKL, a critical factor for osteoclast differentiation^6^ (Fig. 7). Research has demonstrated that TNF-α can enhance RANKL expression on osteoblasts and stromal cells, thereby promoting osteoclastogenesis^6^. The role of IL-17 in osteoclastogenesis remains a subject of debate. Certain studies indicate that IL-17 can promote osteoclast differentiation indirectly by increasing RANKL expression on osteoblasts^309^. However, other research indicates that IL-17 primarily acts indirectly by modulating the inflammatory environment rather than directly affecting OCPs^309^. This highlights the complexity of cytokine interactions in osteoclastogenesis. The activation of the NF-κB pathway is a well-documented mechanism through which inflammatory cytokines induce osteoclast differentiation. NF-κB signaling promotes the expression of key genes required for osteoclastogenesis, such as RANK and c-Fos. Recent studies have shown that inhibiting NF-κB can significantly reduce osteoclast formation in inflammatory conditions^310^.

Pro-inflammatory cytokines such as TNF-α enhance osteoclastogenesis, whereas anti-inflammatory cytokines like IL-10 suppress it, reflecting the intricate balance between inflammatory and anti-inflammatory signals that regulate osteoclast activity^8^. Another study revealed that IL-18, regulated by PTH, is crucial for the anabolic actions of PTH, which involves recruiting and activating OCPs^311^. Li et al.^312^ further expanded on this by showing that PTH stimulates osteoblastic MCP-1 expression, which recruits OCPs, illustrating how osteoblast-derived cytokines directly influence osteoclast formation. The research highlighted that TNF-α promotes osteoclastogenesis, especially when combined with RANKL. This pro-inflammatory cytokine is central to the pathology of bone loss in inflammatory diseases like arthritis^313^. Gu et al.^314^ also showed that during gout remission, osteoclast differentiation pathways are significantly enriched in immune cells, such as monocytes and T cells, emphasizing the role of inflammation in driving osteoclast formation. Zhong et al.^252^ found that adipogenic precursors in bone marrow release cytokines like M-CSF that are essential for osteoclast differentiation, further linking inflammatory signals with increased osteoclastogenesis in inflammatory bone loss. Their study showed that MyD88 activation through inflammatory cytokines, including TNF-α, drives osteoclast formation and promotes bone resorption^137^. Pooja Chawla et al.’s recent findings on breast cancer suggest that the inflammatory microenvironment induced by breast cancer cells via the MRTF/SRF/CTGF axis promotes osteoclastogenesis, providing a strong explanation for the prevalence of osteolytic lesions in breast cancer metastasis^315^. Another study reported that extracellular miRNAs overexpressed in RA joints act as physiological activators of inflammation by stimulating TLR7/8 on human DCs, leading to NF-κB activation and TNF-α secretion^316^.

TGF-β and other signaling pathways play pivotal roles in regulating osteoclast behavior under various physiological and pathological conditions. Zhao’s study demonstrated that TGF-β drives osteoclastogenesis in inflammatory conditions by reprogramming macrophages, while Zhang et al. revealed that EGFR signaling regulates osteoclast activity during endochondral ossification, highlighting the influence of growth factor pathways such as TGF-β and EGFR on osteoclast modulation^317,318^. Additionally, Dai et al.^319^ showed that adipose tissue macrophages secrete osteopontin, which activates osteoclasts, linking systemic metabolic signals to local bone remodeling through cytokine-mediated pathways. These findings underscore the complex regulatory networks involving growth factors and cytokines in osteoclast activity and bone homeostasis.

### Epigenetic regulation in inflammatory osteoclastogenesis

Zhao’s study on EZH2 revealed that this epigenetic modifier silences IRF8, a key negative regulator of osteoclast differentiation. The suppression of IRF8 under inflammatory conditions facilitates excessive osteoclast activity, highlighting the role of epigenetic modifications in inflammatory bone resorption (Table 1)^320^. Cao et al.^321^ showed that Src blockade inhibits VEGF-induced osteoclast activation, suggesting that targeting Src kinase could reduce osteoclast activity in inflammatory bone diseases. Additionally, Hadzic et al.^322^ explored how EGFR signaling regulates osteoclast presence in cartilage, further illustrating how growth factors can influence osteoclastogenesis in inflammation-driven bone resorption.

### Role of miRNAs in inflammation-induced osteoclastogenesis

Emerging evidence suggests that miRNAs are key regulators of osteoclastogenesis in inflammatory environments. For example, miR-146a has been identified as a negative regulator of osteoclast differentiation. Overexpression of miR-146a in OCPs inhibits NF-κB signaling, reducing osteoclast formation^323,324^. Conversely, downregulation of miR-146a in inflammatory conditions promotes osteoclastogenesis (Fig. 5)^323,324^. A novel aspect of osteoclastogenesis regulation involves exosomal miRNAs. Previous research has identified exosomes derived from inflammatory cells as carriers of miRNAs that can modulate osteoclast differentiation. For example, exosomal miR-214 from osteoclasts has been shown to promote osteoclastogenesis and bone resorption in inflammatory conditions^325^.

### Immune cell interactions and co-stimulatory pathways in osteoclastogenesis

The discovery of NF-κB and NFATc1 signaling in osteoclast formation, along with their essential roles in lymph node development, immune response, and inflammatory arthritis, led to the emergence of bone immunology as a scientific field focused on the interactions between bone and immune cells. The maintenance of normal bone morphology is due to the dynamic balance between bone formation and bone resorption, coordinated by various immune cells and bone cells^326^. B cells, under the regulation of T cells, serve as a primary source of basal OPG. In contrast, activated T cells and B cells are major producers of TNF and RANKL during inflammatory conditions, influencing bone resorption through the RANK-RANKL-OPG axis^327–331^. Studies on human B cells demonstrate that CD40 receptor ligation markedly increases OPG mRNA expression, linking OPG production to B-cell–T-cell interactions, as CD40L is abundantly expressed on activated T cells^327–331^. This crosstalk indicates that both B and T cells participate in regulating the basal RANKL:OPG ratio. Furthermore, inhibition of the T-cell co-stimulatory molecule CD28 by CTLA4 directly suppresses RANKL- and TNF-induced osteoclast formation in vitro. Consistently, mice deficient in CD80, CD86, or IDO exhibit elevated osteoclastogenesis and develop an osteopenic phenotype^332,333^. Costimulatory molecules can also activate NFATc1 through ligands binding to immunoglobulin-like receptors such as TREM-2 and OSCAR. Ligands for most receptors on OCP have not been identified, but OSCAR is activated by specific parts of collagen that are exposed during absorption^17^. These receptors recruit adaptor proteins such as FcRγ and DAP12, which undergo ITAM phosphorylation and initiate downstream signaling. Together with co-stimulatory inputs, RANK signaling activates PLCγ and Ca^2+^-CaM pathways, resulting in intracellular Ca^2+^ release, NFATc1 dephosphorylation, and nuclear translocation of calcineurin-NFATc1 complexes^17^. Thus, co-stimulatory signals and RANK synergistically activate NFATc1 to promote osteoclast differentiation and function, positioning NFATc1 as a promising therapeutic target in inflammatory bone diseases^17^.

Cyclosporin A is a calcineurin/NFATc1 inhibitor that has been demonstrated as an immunosuppressive agent to prevent bone loss in mouse RA models and to prevent the activation of NFATc1^17^. Interestingly, activation of NFATc1 by RANK or OSCAR increases the expression of OSCAR on OCP in a positive feedback loop. The expression of OSCAR and RANKL is increased in the synovium of the joints of RA patients, but to date, there have been no reports of OSCAR expression in patients’ inflamed gum tissue^17^. It was also found that NFATc1 can actively regulate the expression of osterix, a transcription factor that plays a fundamental role downstream of Runx2 in osteoblast precursor differentiation^17^. In normal mice, cyclosporin A treatment induces osteoporosis by inhibiting NFATc1-mediated osteoblast formation, suggesting that NFATc1 plays a more critical role in osteoblasts than in osteoclasts during physiological bone remodeling. By contrast, in inflammatory bone diseases, osteoblast activity is suppressed through mechanisms such as TNF- and DKK1-mediated inhibition. Under these conditions, the principal effect of NFATc1 inhibition is the reduction of osteoclast-mediated bone resorption^17^.

### Gene regulation in osteoclasts under inflammatory conditions

Inflammation can also influence osteoclast gene regulation, with certain transcription factors being pivotal. NF-κB and AP-1 are key transcriptional effectors activated by inflammatory cytokines. NF-κB, for instance, is critical for the expression of osteoclast-specific genes and is rapidly activated by RANKL in OCP^334^. Additionally, AP-1 components such as c-Fos are essential for osteoclast differentiation, with c-Fos knockout mice exhibiting severe osteopetrosis due to the lack of functional osteoclasts (Table 1). Other transcription factors like PU.1 and NFATc1 also play crucial roles in osteoclast gene regulation under inflammatory conditions^334^.

Previous study demonstrates that adipose tissue-derived mesenchymal stem cells (ADMSCs) suppress osteoclast differentiation via the secretion of tumor necrosis factor-stimulated gene-6 (TSG-6)^335^. Through co-culture experiments with OCPs, TSG-6 was identified as a critical mediator in reducing TRAP activity and mRNA expression of osteoclast markers under osteoclastogenic conditions^335^. These findings highlight the potential of TSG-6 as a therapeutic target for inflammatory bone diseases (Table 3).

**Table 3:** Epigenetic regulation of osteoclast differentiation.

Epigenetic regulator	Function	Role in osteoclast differentiation	Mechanism	Reference
EZH2	Histone methyltransferase; catalyzes H3K27me3	Promotes osteoclastogenesis by silencing anti-osteoclast genes such as *IRF8*	Methylation-dependent silencing of negative regulators	^195^
ASXL1	Polycomb group protein; interacts with PRC2	Suppresses osteoclast formation by regulating chromatin accessibility	Modifies chromatin landscape to suppress pro-osteoclast genes	^202^
JMJD3 (KDM6B)	Histone demethylase; removes H3K27me3	Promotes osteoclastogenesis by de-repressing osteoclast-specific genes	Demethylation of H3K27 on osteoclast gene promoters	^419,420^
Histone deacetylases (HDACs)	Remove acetyl groups from histones	Generally repress gene expression; HDAC inhibitors suppress osteoclast formation	Histone deacetylation reduces transcription of osteoclast genes	^211,421^
DNMTs	Add methyl groups to DNA, mainly at CpG sites	Regulate osteoclast-specific gene expression	DNA methylation may repress anti-osteoclast genes	^422^
NRF2-UCHL1 axis	Regulates oxidative stress responses and ubiquitin signaling	Inhibits osteoclastogenesis through KEAP1 stabilization	Modulates transcription factors and anti-osteoclast pathways	^97^
miR-19a	miRNA targeting CYLD	Enhances osteoclastogenesis by activating NF-κB and MAPK pathways	Post-transcriptional silencing of CYLD, promoting TRAF6 activation	^95,423^

### Osteomorphs and osteoclast recycling

In McDonald’s novel discovery, osteoclasts were found to recycle via osteomorphs during bone resorption, indicating a unique cellular recycling mechanism that sustains bone resorption in inflammatory conditions^40^. Zhu et al.^336^ supported this by showing that osteoclast activity recovers during drug holidays in osteoporotic rats, suggesting the potential for therapeutic intervention to modulate osteoclast function during treatment.

Inflammatory osteoclastogenesis is driven by pro-inflammatory cytokines like TNF-α and modulated by growth factors and epigenetic changes. Understanding how these factors promote osteoclast activity during inflammation is key to developing therapies for bone diseases like RA and osteoporosis. Targeting these pathways can help prevent excessive bone loss in inflammatory conditions. Developmentally regulated GTP-binding protein 2 (Drg2), a member of the GTPase superfamily, plays a crucial role in osteoclast differentiation and bone mass regulation^337^. Studies have shown that Drg2 knockdown in BMDMs significantly inhibits osteoclast differentiation and reduces Rac1 activation^337^. In vivo, Drg2-deficient mice exhibit increased bone mass with a marked reduction in osteoclast numbers, while osteoblast numbers remain largely unaffected. These findings suggest that Drg2 regulates the formation of mature osteoclasts through Rac1 activation, making it a promising therapeutic target for bone loss-related diseases such as osteoporosis^337^.

The interplay between inflammation and osteoclastogenesis is multifaceted and critical for understanding pathological bone resorption. While substantial progress has been made in elucidating the mechanisms through which inflammatory cytokines and signaling pathways promote osteoclast differentiation, several areas remain controversial. Future research should focus on resolving these discrepancies and exploring novel regulatory mechanisms to develop targeted therapies for inflammatory bone diseases.

### Emerging research and controversies

Studies have highlighted novel regulators of osteoclastogenesis that could serve as therapeutic targets. For example, the chemokine CCL5 (RANTES) has been implicated in promoting osteoclast differentiation by acting as a chemoattractant for OCPs^338^. Another study identified ADAM8 as a key factor in enhancing OCP fusion and osteoclast formation both in vitro and in vivo, suggesting its potential role in inflammatory bone diseases^339^. However, some findings remain controversial or not widely accepted. For instance, while IFN-γ is generally considered a suppressor of osteoclastogenesis, its administration in certain contexts has been shown to paradoxically stimulate bone erosion by promoting T-cell activation and secretion of osteoclastogenic factors^121^. This dichotomy underscores the complexity of cytokine interactions in different inflammatory settings.

## Targeting osteoclast genes for bone lysis and inflammatory diseases

Dysregulation of osteoclast differentiation is a key factor in the development of various bone-related diseases. The regulation of osteoclast activity is pivotal in maintaining bone health and preventing various bone-related diseases. Emerging therapies, such as targeting the Dlk2-Syap1 signaling pathway and utilizing mesenchymal stem cell (MSC)-derived extracellular vesicles, show promise in modulating osteoclast differentiation and activity. These advancements offer potential new treatments for diseases like osteoporosis and osteolytic bone metastasis, highlighting the importance of continued research in this field.

### Osteoporosis

Excessive osteoclast activity underlies conditions such as osteoporosis, characterized by weakened bones and increased fracture risk, and Paget’s disease of bone, which involves abnormal cycles of bone destruction and regrowth, leading to deformity^340^. On the other hand, insufficient osteoclast activity can result in osteopetrosis, where bones become overly dense and prone to fractures^340^. Proper regulation of osteoclast differentiation is essential for maintaining bone health, as excessive osteoclast formation and bone resorption contribute to osteolytic diseases.

Research has highlighted the role of delta-like homolog 2 (Dlk2), a member of the epidermal growth factor (EGF)-like superfamily, in osteoclast regulation^341^. Deletion of Dlk2 significantly impairs osteoclast formation in vitro and results in a high bone mass phenotype in vivo^341^. Dlk2 exerts its effects by interacting with synapse-associated protein 1 (Syap1), which governs the phosphorylation of Akt at Ser473^341^. Loss of Dlk2 disrupts Syap1-mediated activation of Akt, as well as ERK1/2 and p38 signaling pathways, key regulators of osteoclast differentiation^341^. Furthermore, Dlk2 deficiency has been shown to increase bone mass in OVX mice, underscoring the critical role of the Dlk2-Syap1 signaling axis in osteoclast differentiation and the pathogenesis of osteoclast-related bone diseases^341^.

Researchers have shown growing interest in MSC-based therapies as a potential alternative approach for osteoporosis treatment, offering the advantage of reduced side effects^342,343^. Human MSC-derived extracellular vesicles (hMSC-EVs) have been found to inhibit RANKL-induced differentiation of BMDMs into osteoclasts in a dose-dependent manner^342^. This treatment also reduces F-actin ring formation and bone resorption in osteoclasts. Furthermore, EVs decrease RANKL-induced phosphorylation of p38 and JNK and the expression of osteoclastogenesis-related genes in BMDMs treated with RANKL^342^. miRNAs present in these EVs, particularly has-miR122-5p, play a role in regulating osteoclast function. Overexpression of miR122-5p in BMDMs significantly inhibits RANKL-induced osteoclast differentiation, impairs F-actin ring formation, and reduces bone resorption^342^. These findings suggest that hMSC-EVs containing miR122-5p inhibit RANKL-induced osteoclast differentiation by downregulating molecular mechanisms, making them a potential preventive treatment for destructive bone diseases^342^.

Recent research has identified a critical interplay between sensory neuronal signaling and iOCs in the delayed healing of osteoporotic fractures^344^. In OVX mice, impaired signaling of CGRP^+^TrkA^+^ sensory neurons during callus remodeling has been associated with an increased accumulation of Cx3cr1^+^ iOCs within the fracture callus^344^. Conditional deletion of Cx3cr1^+^ iOCs restored CGRP^+^TrkA^+^ neuronal signaling and promoted normal callus remodeling^344^. Mechanistically, Cx3cr1^+^ iOCs secrete Sema3A, which inhibits axonal regeneration of CGRP^+^TrkA^+^ sensory neurons, thereby disrupting nerve–bone crosstalk and impairing effective bone repair^344^. Consistently, analysis of human osteoporotic fracture samples demonstrated an inverse correlation between CGRP^+^TrkA^+^ neuronal activity and Cx3cr1^+^ iOC abundance^344^. Enhancing CGRP^+^TrkA^+^ signaling by inhibiting Cx3cr1^+^ iOCs offers a promising therapeutic avenue for improving fracture healing^344^. These results underscore the pivotal role of sensory neuronal signaling and its regulation by iOCs in osteoporotic fracture repair and callus remodeling.

### Endodontic and periodontal osteolysis and other lytic bone diseases

Dental caries, one of the most common infectious diseases worldwide, affects ~80% of children and most adults. It can lead to endodontic disease, resulting in pulp necrosis, periapical inflammation, bone resorption, severe pain, and tooth loss. Periapical inflammation may also exacerbate inflammation elsewhere in the body^345^. Despite numerous studies attempting to develop treatments for this disease, effective solutions are still urgently needed^274^. An early study employed recombinant adeno-associated virus (AAV)-mediated RNA interference to silence CTSK and ATP6i^346^ expression, thereby targeting osteoclasts and reducing periapical bone resorption in a mouse model^274^. They found that AAV-sh-CTSK impaired osteoclast function in vivo and significantly reduced bacterial infection-stimulated bone resorption by 88%. The reduction in apical lesion area was accompanied by decreased mononuclear leukocyte infiltration and lower expression of inflammatory cytokines. This study demonstrates that AAV-RNAi silencing of CTSK in apical tissue significantly reduces the development of root canal disease, bone destruction, and inflammation of apical lesions^345^. AAV-mediated RNAi knockdown gene therapy can significantly mitigate the severity of root canal disease^345^. Endodontic disease leads to severe bone resorption and tooth loss due to inflammation of periapical tissues. ATP6AP1 (Ac45) is involved in immune responses and inflammatory diseases^347^. AAV-sh-Ac45 reduces bone destruction and inflammation in periapical tissues by impairing cellular acidification and bone resorption. Local delivery of AAV-sh-Ac45 decreases T cell, macrophage, and DC infiltration and protects the periodontal ligament. It also reduces osteoclast formation and proinflammatory cytokine expression^348^. Therefore, Ac45 could be a novel therapeutic target for endodontic disease and other inflammation-related osteolytic conditions^348^.

Dysregulated osteoclast activity underlies a spectrum of lytic bone diseases characterized by focal bone resorption and structural instability. V-ATPase–CTSK resorptive machinery revealed a conserved molecular program that mediates matrix degradation and promotes cartilage–bone crosstalk through angiogenesis and sensory innervation, thereby contributing to pain and progressive bone destruction^280^. Upregulation of V-ATPase subunits and CTSK amplifies matrix turnover and osteolysis under both degenerative and neoplastic conditions^280^. Moreover, osteoclast-targeted bone sialoprotein (BSP) overexpression was shown to accelerate breast cancer bone metastasis, highlighting osteoclasts as active participants in metastatic niche formation and focal osteolytic lesions^349^. These findings extend the significance of osteoclast biology from joint degeneration to malignant and systemic lytic disorders, underscoring osteoclast-centered pathways as convergent mediators of pathological bone loss across diverse disease contexts.

### RA and inflammatory bone loss

RA is a chronic autoimmune disease characterized by persistent synovial inflammation and progressive joint destruction, in which osteoclasts act as the central effectors of inflammatory bone erosion. In RA, activated immune and stromal cells secrete high levels of RANKL, TNF-α, IL-1β, and IL-6, which drive excessive osteoclastogenesis through amplification of NF-κB, MAPK, and JAK-STAT signaling. This aberrant cytokine milieu overrides physiological restraints, promoting osteoclast differentiation within inflamed pannus tissue and accelerating subchondral bone loss. Recent findings from Li Lab have identified several intrinsic regulators that fine-tune this process. Gα_13_ functions as a critical brake on osteoclastogenesis; its loss enhances RhoA-Akt-GSK3β-NFATc1 signaling and exacerbates inflammatory osteolysis, whereas restoration of Gα_13_ constrains erosions^246^. TANK, a RANKL-induced adaptor, provides negative feedback to limit NF-κB signaling and osteoclast survival, thereby mitigating inflammatory bone destruction^58^. In contrast, RGS12 promotes osteoclast differentiation and pathological bone loss, as its deletion increases bone mass and dampens Ca^2+^/ERK signaling^120^. Additionally, IFT80 acts as a negative regulator by destabilizing TRAF6, suppressing excessive osteoclast formation and protecting against erosion^60^.

A recent study on RA investigated the role of aconitate decarboxylase 1 (Acod1) and its metabolite itaconate in regulating osteoclast differentiation and bone loss in inflammatory arthritis^350^. Itaconate levels in peripheral blood mononuclear cells from patients with RA were found to inversely correlate with disease activity, suggesting a protective function. Acod1-deficient mice exhibited increased osteoclast numbers and exacerbated bone erosion in experimental arthritis models, emphasizing its role in suppressing osteoclastogenesis^350^. Treatment with 4-octyl-itaconate (4-OI), an itaconate derivative, alleviated inflammatory bone loss in vivo and inhibited osteoclast differentiation in human and murine cells in vitro. Mechanistically, Acod1 inhibited osteoclast differentiation by suppressing succinate dehydrogenase activity, reducing ROS production^350^. Itaconate further blocked HIF1α-mediated aerobic glycolysis, a critical metabolic pathway in osteoclast development. RNA sequencing revealed that Acod1-induced metabolic changes regulate osteoclast transcriptomes^350^. Using CRISPR/Cas9, HIF1α was confirmed as a key mediator in Acod1’s metabolic regulation of osteoclastogenesis. These findings demonstrate that Acod1 and itaconate act as crucial regulators of osteoclast differentiation and inflammatory bone loss^350^. This work suggests that targeting Acod1 or utilizing itaconate derivatives could provide novel therapeutic strategies for managing RA-associated bone degradation^350^. A recent study also found that MYO6 deficiency attenuates arthritis development and bone destruction in CIA mice and impairs osteoclast differentiation by inhibiting NFATc1 induction^351^. MYO6 is essential for podosome organization by regulating the FAK/AKT and integrin-β3/Src pathways^351^. MYO6 also mediates endosomal trafficking by regulating the expression of Rab5 and GM130^351^. This may affect the maintenance and function of the ruffled border and the regulation of osteoclast autophagy. Besides, research also demonstrated that HSP90 promotes osteoclast differentiation and exacerbates bone destruction in RA by activating TRAF6/NFATc1 signaling. Interfering with HSP90 may be a promising strategy for discovering drugs against bone destruction in arthritis^352^.

### Osteoarthritis

Osteoarthritis (OA) represents a degenerative joint disorder characterized not only by articular cartilage breakdown but also by abnormal remodeling of the subchondral bone, where osteoclasts play an increasingly recognized pathogenic role^353^. Aberrant activation of osteoclasts in the osteochondral unit disrupts bone–cartilage coupling and promotes mechanical and biochemical microenvironmental changes that accelerate cartilage degeneration^353^. RGS12 facilitates osteoclast differentiation by acting upstream of the Ca^2+^/ERK-NFATc1 pathway, coupling excessive osteoclastogenesis with subchondral remodeling and OA-like bone alterations^354^. In contrast, Gα_13_ serves as an intrinsic brake on osteoclastogenesis by restraining RhoA-Akt-GSK3β-NFATc1 signaling, thereby preserving osteochondral coupling and attenuating bone erosion during OA progression^246^. These findings suggest that osteoclast dysfunction contributes directly to the pathogenesis of OA, not merely as a secondary response to cartilage loss but as a central driver of osteochondral uncoupling and inflammatory bone turnover.

## Perspectives and future studies

Osteoclasts are central to bone homeostasis, functioning not only as bone-resorbing cells but also as dynamic regulators of immune responses, metabolism, and intercellular communication within bone and beyond. While the transcriptional, cytokine, and signaling pathways governing osteoclastogenesis are increasingly well-characterized, many aspects of osteoclast biology remain incompletely understood. Below, we highlight major unresolved questions and future research direcions.

### Distinguishing physiological vs pathological bone loss

A key challenge in the field is to clearly delineate physiological bone remodeling from pathological bone destruction. Physiological bone resorption is essential for maintaining skeletal strength, remodeling microdamaged bone, and adapting to mechanical load. It involves balanced M-CSF and RANKL signaling, producing short-lived osteoclasts that resorb bone locally and transiently. Pathological bone loss, by contrast, results from dysregulated infammatory and hormonal cues, leading to prolonged osteoclast survival and hyperactivation. Future research may focus on: (1) Define distinct molecular signatures distinguishing physiological vs pathological osteoclasts (e.g., iOC subsets). (2) Identify unique precursor populations or tissue-resident macrophage contributions to disease-associated osteoclasts. (3) Explore sex hormone- and aging-dependent shifts in osteoclast lineage commitment and survival.

### Osteoclast heterogeneity across anatomical sites

Osteoclasts exhibit remarkable anatomical and developmental heterogeneity. Calvarial, long bone, and alveolar osteoclasts differ in origin, signaling responsiveness, and resorptive behavior. Future research may focus on: (1) Mapping site-specific osteoclast transcriptomes and epigenomes using scRNA-seq, ATAC-seq, and spatial transcriptomics. (2) Investigating osteoclast–osteoblast coupling and matrix composition-dependent differentiation at site-specific niches. (3) Understanding local biomechanical and metabolic influences (e.g., mechanical loading in long bones vs craniofacial bone regeneration).

### Osteoclast heterogeneity under inflammatory conditions

Inflammatory stimuli reprogram osteoclasts into phenotypically distinct subtypes as “iOCs” that exhibit high resorptive activity and secrete immunoregulatory cytokines. Future research may focus on: (1) Defining transcriptional regulators that mediate inflammatory reprogramming. (2) Investigating interactions with T cells, B cells, and myeloid cells, which may determine osteoclast fate and activity in diseases such as RA, periodontitis, and bone metastasis. (3) Using multi-omics and lineage tracing to distinguish iOCs from classical bone-resorbing and coupling osteoclasts. (4) Exploring osteoclast-derived cytokines that modulate immune tolerance and osteoimmunology.

### Metabolic regulation and systemic crosstalk

Osteoclasts are metabolically active cells with tightly regulated energy demands. Mitochondrial function, glycolysis, and lipid oxidation dynamically change during differentiation and activation. Future research may focus on: (1) Metabolic reprogramming in osteoclastogenesis. (2) Mitochondrial dynamics (fusion/fission) and ROS regulation in bone resorption. (3) Crosstalk with systemic metabolism, how osteoclast-derived factors influence glucose and lipid metabolism, and how obesity or diabetes modulate osteoclast function. (4) Investigating hormonal regulation of osteoclast activity, as well as the impact of aging and menopause.

### Epigenetic and chromatin landscape of osteoclasts

Epigenetic regulation plays a pivotal role in determining osteoclast lineage commitment, differentiation, and functional plasticity. Beyond classical transcription factors such as NFATc1 and c-Fos, chromatin remodeling and non-coding RNAs fine-tune osteoclast gene expression and responsiveness to microenvironmental cues. Key aspects and future directions include: (1) Histone modifications: histone acetylation by histone acetyltransferases (HATs) promotes expression of osteoclast genes like *Ctsk* and *Acp5*, whereas deacetylation by HDACs suppresses differentiation. (2) Histone methyltransferases regulate chromatin accessibility and lineage fidelity; targeting these enzymes may modulate pathological osteoclast activity. (3) DNA methylation: differential methylation at *RANK*, *NFATc1*, and *DC-STAMP* promoters affect osteoclast formation during aging and inflammation. Future work should map single-cell DNA methylomes to identify epigenetically distinct osteoclast subsets. (4) Chromatin remodeling complexes: SWI/SNF (BAF) and NuRD complexes alter nucleosome positioning to facilitate or repress osteoclast-specific enhancer activation. Mutations in chromatin regulators may contribute to skeletal dysplasias and aberrant bone resorption. (5) Non-coding RNAs: miRNAs and lncRNAs (lnc-Ctsk, lnc-Nfatc1) serve as fine modulators of osteoclastogenesis. circRNAs and piRNAs are emerging as new layers of post-transcriptional regulation influencing RANKL-RANK signaling and cytoskeletal organization. (6) Therapeutic perspectives: small-molecule HDAC inhibitors, BET bromodomain inhibitors, and DNA methylation modulators represent promising approaches to selectively dampen pathological osteoclast activation. Integration of multi-omics and single-cell epigenomics will clarify how transcriptional, metabolic, and signaling networks converge on chromatin to orchestrate osteoclast behavior.

### Mechanobiology and osteoclast function

Mechanical stress and microenvironmental stiffness profoundly influence osteoclast activation. Emerging directions may include: (1) Mechanotransduction via Piezo1/2, integrins, and cytoskeletal remodeling. (2) 3D imaging of osteoclast mechanosensitivity using advanced intravital microscopy. (3) Exploring how mechanical unloading (e.g., microgravity or immobilization) promotes osteoclast-mediated bone loss.

### Osteoclast senescence and aging

Aging alters osteoclast function through cellular senescence, oxidative stress, and altered cytokine milieu. Future research may focus on: (1) Do senescent osteoclasts or precursors exist, and how do they contribute to age-related bone loss? (2) Can senolytic therapies or autophagy modulators rejuvenate osteoclast function? (3) How do DNA damage response pathways influence osteoclast survival in aged bone?

### Artificial intelligence and multi-omics integration

Artificial intelligence (AI) and machine learning will transform osteoclast biology by enabling: (1) Predictive modeling of osteoclast differentiation from transcriptomic and proteomic datasets. (2) Integration of multi-omics data (genomics, proteomics, metabolomics, spatial transcriptomics) to identify master regulators of pathological resorption. (3) AI-assisted drug screening and in silico modeling to simulate osteoclast–immune–bone interactions.

### Organoids and organ-on-chip systems

Advanced 3D bone organoids and organ-on-chip systems provide human-relevant models to study osteoclast function in physiologically realistic microenvironments. These platforms replicate bone remodeling dynamics, immune interactions, and mechanical cues more accurately than conventional cultures or animal models. They enable real-time analysis of osteoclast differentiation and drug responses under controlled conditions. Integration with AI and multi-omics will further enhance their predictive and translational potential in bone disease research.

### Translational and therapeutic frontiers

Despite progress in anti-resorptive therapies (bisphosphonates, denosumab), challenges remain in selectively targeting pathological osteoclasts while preserving normal remodeling. Future approaches may focus on: (1) Osteoclast-specific delivery systems (antibody-conjugated nanoparticles, liposomes). (2) Targeting osteoclast–immune interactions in bone metastasis and inflammatory diseases. (3) Exploring human-based 3D bone–immune organoids and organ-on-chip systems to model osteoclast behavior under physiological flow and mechanical loading.

## Summary

Osteoclasts are multifaceted, plastic, and context-dependent cells that bridge bone, immune, and metabolic systems. Integrating multi-omics, spatial biology, structural bioinformatics, and AI-driven discovery will reshape our understanding of osteoclast heterogeneity and open new horizons for precision medicine in bone diseases.
